# Analogous assembly mechanisms and functional guilds govern prokaryotic communities in mangrove ecosystems of China and South America

**DOI:** 10.1128/spectrum.01577-23

**Published:** 2023-09-05

**Authors:** Huan Du, Jie Pan, Cuijing Zhang, Xilan Yang, Cheng Wang, Xiaolan Lin, Jinhui Li, Wan Liu, Haokui Zhou, Xiaoli Yu, Shuming Mo, Guoqing Zhang, Guoping Zhao, Wu Qu, Chengjian Jiang, Yun Tian, Zhili He, Yang Liu, Meng Li

**Affiliations:** 1 Archaeal Biology Center, Institute for Advanced Study, Shenzhen University, Shenzhen, China; 2 Shenzhen Key Laboratory of Marine Microbiome Engineering, Institute for Advanced Study, Shenzhen University, Shenzhen, China; 3 Shenzhen Xbiome Biotech Co. Ltd., Shenzhen, China; 4 Shenzhen Key Laboratory of Synthetic Genomics, Guangdong Provincial Key Laboratory of Synthetic Genomics, CAS Key Laboratory of Quantitative Engineering Biology, Shenzhen Institute of Synthetic Biology, Shenzhen, China; 5 Institute of Synthetic Biology, Shenzhen Institutes of Advanced Technology, Chinese Academy of Sciences, Shenzhen, China; 6 Southern Marine Science and Engineering Guangdong Laboratory (Zhuhai), Zhuhai, China; 7 State Key Laboratory for Biocontrol, Environmental Microbiomics Research Center, School of Environmental Science and Engineering, Sun Yat-sen University, Guangzhou, China; 8 Key Laboratory of the Ministry of Education for Coastal and Wetland Ecosystems, School of Life Sciences, Xiamen University, Xiamen, China; 9 National Engineering Research Center for Non-Food Biorefinery, Guangxi Research Center for Biological Science and Technology, Guangxi Academy of Sciences, Nanning, China; 10 National Genomics Data Center& Bio-Med Big Data Center, CAS Key Laboratory of Computational Biology, Shanghai Institute of Nutrition and Health, University of Chinese Academy of Sciences, Chinese Academy of Science, Shanghai, China; 11 Hangzhou Institute for Advanced Study, University of Chinese Academy of Sciences, Hangzhou, China; 12 Marine Science and Technology College, Zhejiang Ocean University, Zhoushan, China; Hong Kong University of Science and Technology, Hong Kong

**Keywords:** microbial composition, assembly pattern, keystone taxa, key functional guilds, mangrove microbiome

## Abstract

As an important coastal “blue carbon sink,” mangrove ecosystems contain microbial communities with an as-yet-unknown high species diversity. Exploring the assemblage and structure of sediment microbial communities therein can aid in a better understanding of their ecosystem functioning, such as carbon sequestration and other biogeochemical cycles in mangrove wetlands. However, compared to other biomes, the study of mangrove sediment microbiomes is limited, especially in diverse mangrove ecosystems at a large spatial scale, which may harbor microbial communities with distinct compositions and functioning. Here, we analyzed 380 sediment samples from 13 and 8 representative mangrove ecosystems, respectively, in China and South America and compared their microbial features. Although the microbial community compositions exhibited strong distinctions, the community assemblage in the two locations followed analogous patterns: the assemblages of the entire community, abundant taxa, rare taxa, and generalists were predominantly driven by stochastic processes with significant distance-decay patterns, while the assembly of specialists was more likely related to the behaviors of other organisms in or surrounding the mangrove ecosystems. In addition, co-occurrence and topological network analysis of mangrove sediment microbiomes underlined the dominance of sulfate-reducing prokaryotes in both the regions. Moreover, we found that more than 70% of the keystone and hub taxa were sulfate-reducing prokaryotes, implying their important roles in maintaining the linkage and stability of the mangrove sediment microbial communities. This study fills a gap in the large-scale analysis of microbiome features covering distantly located and diverse mangrove ecosystems. Here, we propose a suggestion to the Mangrove Microbiome Initiative that 16S rRNA sequencing protocols should be standardized with a unified primer to facilitate the global-scale analysis of mangrove microbiomes and further comparisons with the reference data sets from other biomes.

**Importance:**

Mangrove wetlands are important ecosystems possessing valuable ecological functions for carbon storage, species diversity maintenance, and coastline stabilization. These functions are greatly driven or supported by microorganisms that make essential contributions to biogeochemical cycles in mangrove ecosystems. The mechanisms governing the microbial community assembly, structure, and functions are vital to microbial ecology but remain unclear. Moreover, studying these mechanisms of mangrove microbiomes at a large spatial scale can provide a more comprehensive insight into their universal features and can help untangle microbial interaction patterns and microbiome functions. In this study, we compared the mangrove microbiomes in a large spatial range and found that the assembly patterns and key functional guilds of the Chinese and South American mangrove microbiomes were analogous. The entire communities exhibited significant distance-decay patterns and were strongly governed by stochastic processes, while the assemblage of specialists may be merely associated with the behaviors of the organisms in mangrove ecosystems. Furthermore, our results highlight the dominance of sulfate-reducing prokaryotes in mangrove microbiomes and their key roles in maintaining the stability of community structure and functions.

## INTRODUCTION

Mangrove wetlands are ecologically important ecosystems widespread along tropical and subtropical coastlines. With only 0.5% of the coastal area, they contribute 10–15% of the total carbon storage of coastal sediments and input 10–11% of the particulate terrestrial carbon to the ocean, making the mangrove ecosystem an important “blue carbon sink” (1, 2). The unique geography as intertidal regions linking land and ocean results in specific ecological features in mangrove ecosystems, such as high nutrient availability, high salinity, low oxygen content, and strong redox potential (3). Such settings create specific biotope, leading to the high diversity and abundance of microbial communities, which play an indispensable role in regulating biochemical processes and hence in maintaining the high productivity of mangrove ecosystems (4 – 7). Despite the important contributions to carbon economy and biogeochemical cycling, the study of microbiomes in mangrove ecosystems, especially the global-scale analysis, was limited compared with other biomes, such as terrestrial, aquatic, or human microbiomes (8 – 13). In this context, the Mangrove Microbiome Initiative, an international network of researchers, was announced to advance mangrove microbial research (14).

Disentangling the assembly mechanism of microbial communities is a central but long-standing challenge in ecological research (15). Two main theories have emerged for explaining community assembly processes. Neutral theory assumes that community assemblies are driven by stochastic factors such as birth, death, dispersal, immigration, ecological drift, and speciation, whereas niche theory hypothesizes that deterministic processes governed by species traits, interspecies interactions, and environmental conditions determine the community composition (16 – 18). Microbial community assemblages in mangrove ecosystems are considered simultaneously following the two theories, whereas their relative importance is controversial in different local mangrove regions. For example, microbial communities were predominantly driven by stochastic processes in four mangrove regions in Fujian province in China (19), while deterministic processes were more important in six mangrove regions from Zhejiang to Hainan provinces in China (4, 19).

According to previous studies, different traits of microbial groups in the same community are assembled by distinct mechanisms, which may be one of the reasons leading to the complicated dynamics of microbial community assemblies (20, 21). For example, generalists that have a relatively wide habitat range were reported to be mainly driven by neutral processes, while specialists that tend to be restricted in specific habitats tended to follow niche theory (20, 22, 23). The assemblages of abundant taxa and rare taxa distinguished by their relative abundance were also reported to be governed by different variables (24). In addition, although abundant taxa are generally considered important for community structure and function, some rare taxa may also act as key taxa in community structures as well as in ecological functions (25, 26). Thus, analyzing the features of microbial groups with different traits may be critical for investigating the assembly mechanisms and structures of entire communities.

Apart from the impacts of microbial groups with different traits on community assembly patterns, mangrove ecosystems in different areas may present distinct community compositions and functions due to distant locations and variations in environmental conditions (27, 28). However, most studies on microbiomes in mangroves were limited to a relatively small spatial range without intercontinental comparisons (14). Since different continents exhibit isolation in environments and spatial distance, the intercontinental comparison would be a good way to study whether spatial partitioning affects the structures of microbial communities in mangrove ecosystems. We observed some distinctions in mangrove microbial community structures on different continents based on previous studies. For example, in some mangroves in China, the most abundant bacterial and archaeal phyla were, respectively, *Proteobacteria*, *Chloroflexi, Bacteroidetes*, and *Thaumarchaeota* (19), while *Thermoplasmata* (phylum *Euryarchaeota*) and *Campylobacteria* (phylum *Epsilonbacteraeota*) were the most prevalent classes following *Deltaproteobacteria* in part of Brazilian mangroves (29). However, there is a lack of systematic studies for the intercontinental comparison of the microbial community assembly and structures in mangrove ecosystems.

Network analysis-based approaches have proven powerful for providing comprehensive insights into complex microbiome profiles from large-scale microbial community studies (30, 31). For example, the tmap framework, one of the topological network analysis approaches, can transform high-dimensional microbiome data into a network and map the enrichment level of target variables (e.g., microbiome features or metadata) into the network to capture subtle and non-linear associations of taxa and habitat features (32). Another approach is co-occurrence network analysis, which has been widely used to explore non-random co-occurrence patterns in microbial communities and positive or negative correlations of microbes across samples (33 – 35). By evaluating the topological parameters of the co-occurrence network (e.g., closeness centrality, betweenness centrality, or mean degree), keystone taxa can be statistically identified, which are considered as important members or guilds for driving the community structure and functioning irrespective of abundances (36). Untangling pivotal taxa is an important step toward understanding and ensuring community structures and functioning (36). For example, *Anaerolinea* of *Chloroflexi*, identified as a keystone taxon, was detected as an important biogeochemical linker in Yunxiao mangrove microbiomes (5).

Accordingly, in this study, we aimed to address the following questions through a large-scale mangrove sediment microbial community analysis: (i) Would microbial groups with distinct traits follow different community assembly processes? How do the processes and environmental factors affect community assemblages? (ii) What are the key prokaryotic taxa and functional guilds in mangrove ecosystems? (iii) Would microbial communities in different mangrove ecosystems exhibit similar or distinct features in assembly patterns, community compositions, and key functional guilds? To this end, we performed a large-scale analysis of 16S rRNA gene amplicon sequencing data sets including 380 sediment samples from 13 mangroves in China and eight mangroves in South America and compared their microbial features in these two regions. The effects of stochastic and deterministic processes on community assemblages were evaluated for microbial groups with different traits, including all taxa, abundant taxa, rare taxa, generalists, and specialists. Then, the putative keystone taxa and prominent functional guilds were identified using co-occurrence network analysis combined with functional gene predictions. Moreover, the tmap framework (32) was applied to identify enrichment levels and associations of locations, taxa, and functional genes, which provided guidance for the observed results related to community compositions and assemblages.

## RESULTS

### Assembly patterns of sediment prokaryotic communities in mangrove ecosystems

Overall, after combining our data with the data retrieved from previous studies (29, 37, 38), 279 sediment samples from 13 mangroves in China and 101 sediment samples from 8 mangroves in South America sequenced with the 515F-806R primer pair were analyzed (Fig. S1; Dataset S1, sheet1). After trimming and picking operational taxonomic units (OTUs), a total of 19,548 OTUs were obtained from the Chinese mangrove sediment samples. Four special groups of OTUs with different traits, including 385 abundant OTUs (2.0% of all OTUs), 19,163 rare OTUs (98.0% of all OTUs), 1,642 generalists OTUs (8.4% of all OTUs), and 2,658 specialists OTUs (13.6% of all OTUs), were identified. For the South American samples, a total of 12,891 OTUs were obtained and then 168 abundant OTUs (1.3% of all OTUs), 12,723 rare OTUs (98.7% of all OTUs), 1,054 generalists OTUs (8.2% of all OTUs), and 1,481 specialists OTUs (11.5% of all OTUs) were identified, respectively.

**Fig 1:**
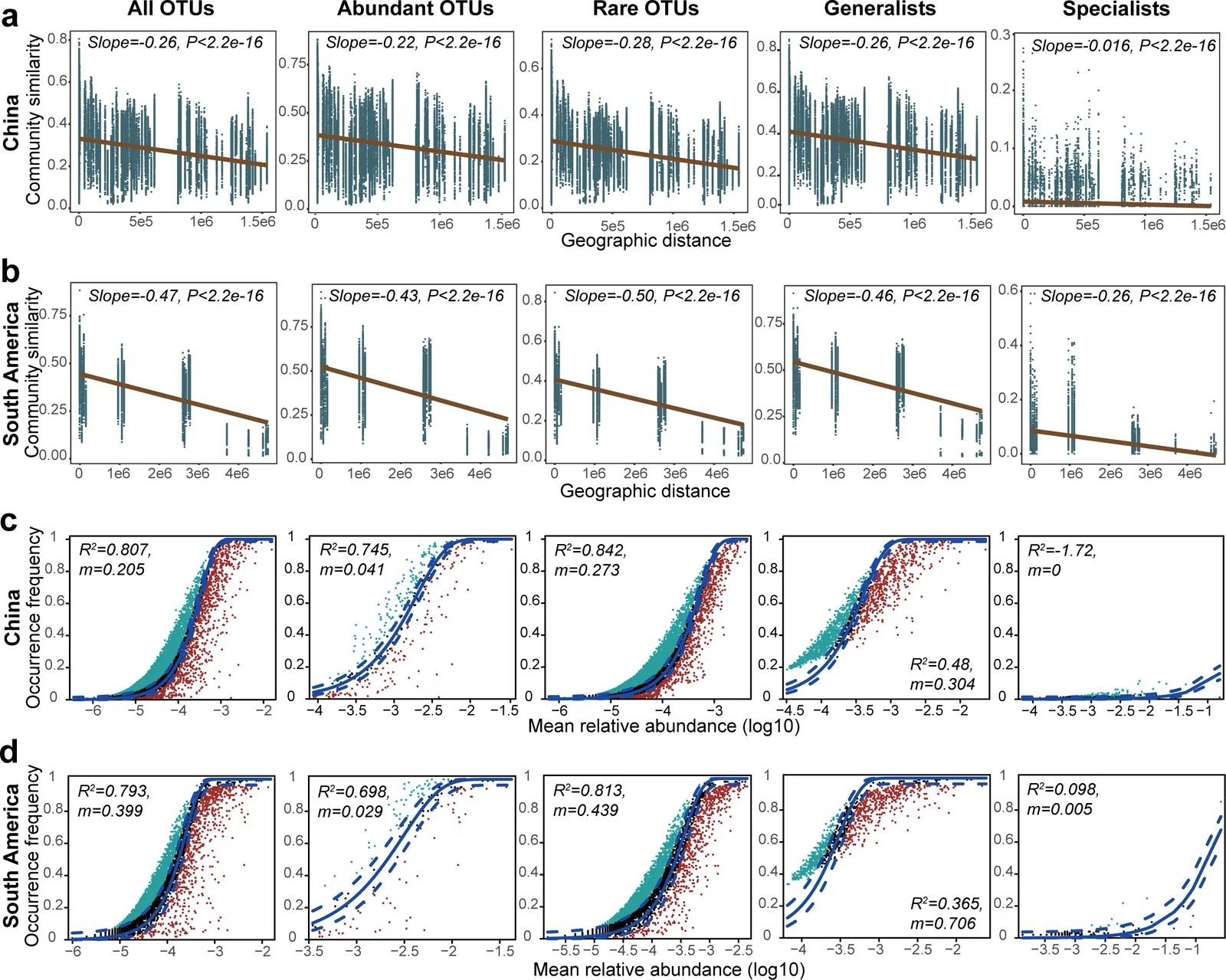
Impacts of stochastic processes on the assemblage of five prokaryotic (sub-)communities with different traits composed of all OTUs, abundant OTUs, rare OTUs, generalists, and specialists. Distance-decay relationships of the five (sub-)communities among diverse mangrove ecosystems in China (**a**) and in South America (**b**). *Slope*, distance-decay slope (i.e., rate at which similarity decreases with distance); *P*, significance of the model. Fitting neutral community model (NCM) to the five (sub-)communities in Chinese (**c**) and South American (**d**) mangrove ecosystems. Solid blue lines denote the best fit of the NCM; dashed blue lines represent 95% confidence intervals around the best-fit neutral model. *R* ^2^, goodness of fit to the model; *m*, estimated immigration rate.

First, significant (*P* < 0.05) linear distance-decay relationships, which reflect the increasing changes in community compositions along the spatial distance, were observed for all the five groups of OTUs in both Chinese and South American samples (Fig. 1a and b). For the Chinese samples, the slopes of the distance-decay pattern for all OTUs, abundant OTUs, rare OTUs, and generalists were similar (from −0.22 to −0.28), while specialists possessed a much gentler slope than the other groups (−0.016). These results indicated that the distance-decay relationships for specialists in Chinese mangrove ecosystems were weaker than those of any other groups. For the South American samples, steeper slopes were obtained for all OTUs, abundant OTUs, rare OTUs, and generalists (from −0.5 to −0.43) than for specialists (−0.26). As in Chinese mangrove sediments, specialists in South American mangrove ecosystems also exhibited weaker distance-decay relationships than the other groups.

The neutral community model (NCM) estimated a large part of community variations (*R* ^2^ ranged from 0.37 to 0.84) for all OTUs, abundant OTUs, rare OTUs, and generalists in both Chinese and South American samples (Fig. 1c and d), suggesting the great contribution of stochastic processes to microbial community assemblies in mangrove sediments. The distinction was that all OTUs (*R* ^2^ ≈ 0.8), abundant OTUs (*R* ^2^ ≈ 0.7), and rare OTUs (*R* ^2^ ≈ 0.8) were more consistent with the neutral process of dispersal and ecological drift than the generalists (*R* ^2^ ≈ 0.4). However, the NCM showed that there was almost no relationship between occurrence frequency and abundance variations of the specialists (*R* ^2^ < 0 for Chinese samples and *R* ^2^ = 0.098 for South American samples), indicating that the assembly pattern of specialists was barely governed by stochastic processes. Besides, the estimated migration rate (*m*), which measures the effect of dispersal on community assembly, was higher for generalists than for any other groups. In addition, the dispersal of abundant OTUs (*m* < 0.05) was more limited than that of all OTUs, rare OTUs, and generalists, implying the greater dispersal limitation of abundant OTUs.

**Fig 2:**
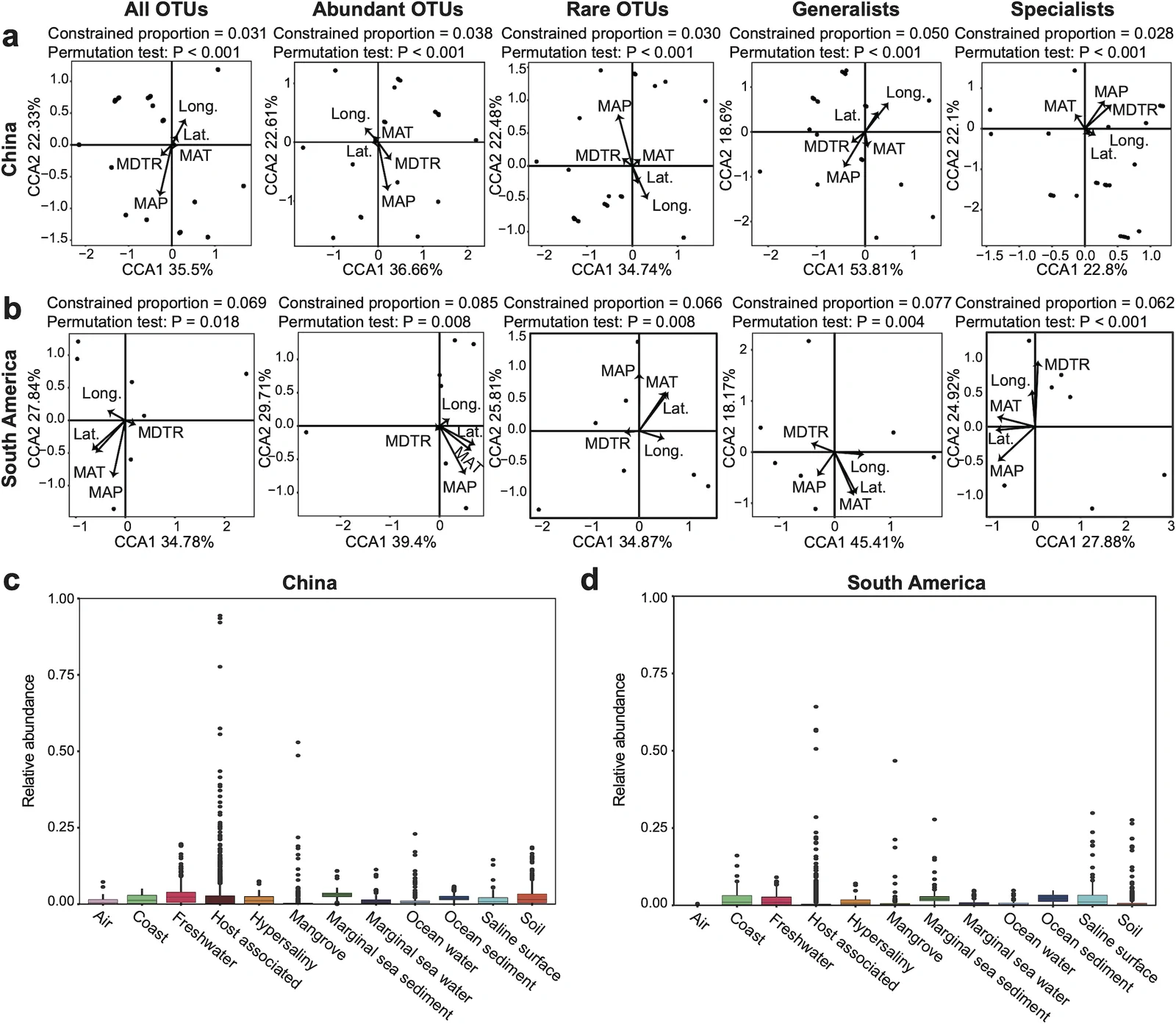
Impact of deterministic processes on community assemblages. Canonical correspondence analysis (CCA) of the relationship between environmental factors and the composition of five prokaryotic sub-communities (i.e., all OTUs, abundant OTUs, rare OTUs, generalists, and specialists) in Chinese (**a**) and South American (**b**) mangrove ecosystems. Arrows represent quantitative environmental factors, while dots represent samples from Chinese or South American mangrove ecosystems. *Long*., longitude; *Lat*., latitude; *MAT*, mean annual temperature; *MAP*, mean annual precipitation; *MDTR*, mean daily temperature range. Boxplot of the relative abundances of specialists identified in China (**c**) and South America (**d**) mangrove sediments in other biomes.

The CCA results showed that longitude, mean annual precipitation (MAP), and mean daily temperature range (MDTR) significantly (*P* < 0.05) affected all OTUs and abundant OTUs of the Chinese mangrove microbiota (Fig. 2a; Dataset S1, sheet2). Apart from these three parameters, latitude also had a relatively strong impact on rare OTUs. Generalists were affected by all five parameters, while only MAP and MDTR were noteworthy for influencing specialists. The CCA of the South American mangrove microbial communities presented some distinctions from those of the communities in Chinese mangrove ecosystems (Fig. 2b). Latitude, MAP, and mean annual temperature (MAT) significantly (*P* < 0.05) affected all OTUs and abundant OTUs (Fig. 2b; Dataset S1, sheet2). Apart from these three parameters, longitude was also a remarkable factor influencing rare OTUs. MDTR was an additional factor that affected specialists. For generalists, all five parameters significantly (*P* < 0.05) affected the community. Even though environmental and spatial factors significantly (*P* < 0.05) affected microbial communities, the constrained proportions of CCA results ranged from 0.028 to 0.085 (Fig. 2a and b), suggesting that only a small proportion of microbial community variation could be explained by the environmental and spatial factors analyzed in this study.

According to the aforementioned analysis, we observed that neither the environmental factors analyzed in this study nor the neutral processes were the major factors shaping the assembly of specialists. To explore the factors affecting the assembly patterns of specialists, the OTU table of the Chinese and South American mangrove ecosystems was, respectively, merged with the EMP OTU table containing 27,751 samples from diverse environments. Then, the abundances of these specialists were calculated in other environments (Fig. 2c and d). For the Chinese samples, the mean abundance of these OTUs was all low (<0.05), except in 28 host-associated samples in which the relative abundances of these OTUs were higher than 0.2 (Fig. 2c). Among the 28 host-associated samples, there were 12 kelp forest samples, 9 honeybee corpus, and 5 human-associated samples (Dataset S1, sheet3). As with the Chinese samples, the mean abundances of specialists in other environments were all low (<0.05), except in four host-associated samples in which specialists had extremely high abundances (>0.5) (Fig. 2d; Dataset S1, sheet3). All four samples were related to aquatic animals. Moreover, the abundance of OTUs in the other four groups (i.e., all OTUs, abundant OTUs, rare OTUs, and generalists) was low in the host-associated samples (Fig. S2). Thus, we inferred that the relationship between the specialists and the behaviors of the organisms in or surrounding the mangrove ecosystems may be a non-ignorable factor affecting the assembly pattern of specialists. To further deduce this relationship, we identified specialists OTUs and found that some of them have been reported to be host-associated in previous studies (details in the Discussion section).

### Taxonomic abundance and network analysis of sediment prokaryotic communities in mangrove ecosystems

**Fig 3:**
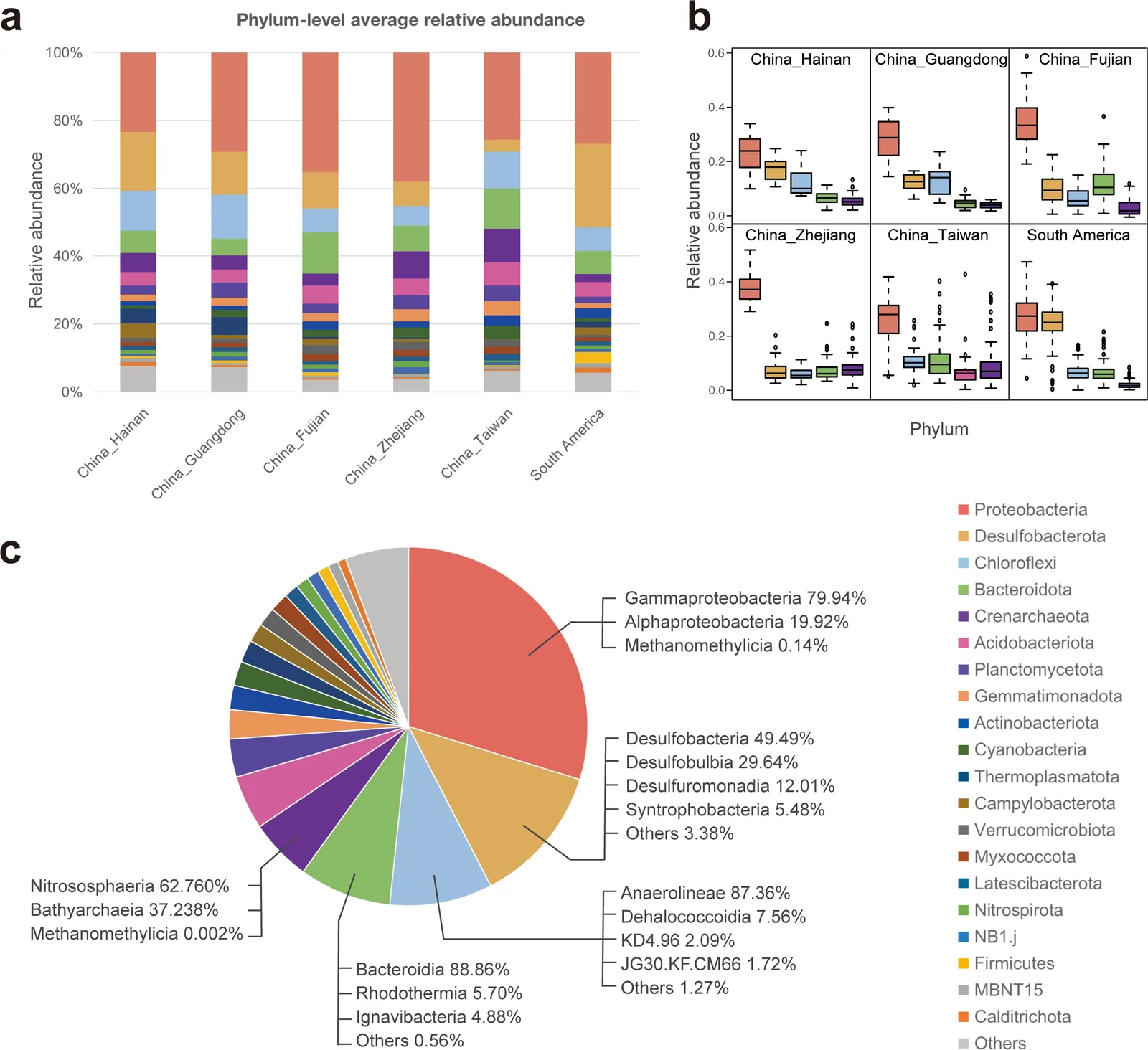
Microbial community compositions in mangrove sediments. (**a**) Mean relative abundances of dominant phyla in mangrove sediments in different locations; (**b**) Boxplot of the relative abundances of the four top abundant bacterial phyla and one top abundant archaeal phylum in different locations; (**c**) Percentages of sequences at the level of class assigned in the top five phyla averaged in all mangrove sediment samples. Colors denote different phyla in all three figures.

The PCoA clearly revealed distinctions in community composition between the Chinese and South American mangrove microbiomes (Fig. S3), whereas according to the relative abundances, the predominant phyla were similar in both locations. *Proteobacteria*, *Desulfobacterota*, *Chloroflexi*, and *Bacteroidota* were simultaneously within the top four abundant bacterial phyla in all the sampling locations, except in Taiwan, while *Crenarchaeota* was dominant in archaeal phyla (Fig. 3a and b; Dataset S1, sheet4). In Taiwan mangrove sediments, the relative abundance of *Desulfobacterota* decreased, and *Acidobacteriota* became one of the top four abundant bacterial phyla, replacing *Desulfobacterota*. The absolutely predominant phylum in Chinese mangrove sediments was *Proteobacteria*, while *Desulfobacterota* was as abundant as *Proteobacteria* in the South American samples. In the phylum of *Proteobacteria*, *Gammaproteobacteria* was the most prominent class, occupying an average of 79.94% of the abundance of *Proteobacteria* in all samples, while *Alphaproteobacteria* was the second abundant class, with an average abundance of 19.92% (Fig. 3c; Dataset S1, sheet4). In *Desulfobacterota*, four classes (*Desulfobacteria*, *Desulfobulbia*, *Desulfuromonadia*, and *Syntrophobacteria*) were dominant, occupying an average of 96.62% of the abundance of *Desulfobacterota* in all samples. *Crenarchaeota* was mainly composed of *Nitrososphaeria* (62.76%) and *Bathyarchaeia* (37.24%) (Fig. 3c).

**Fig 4:**
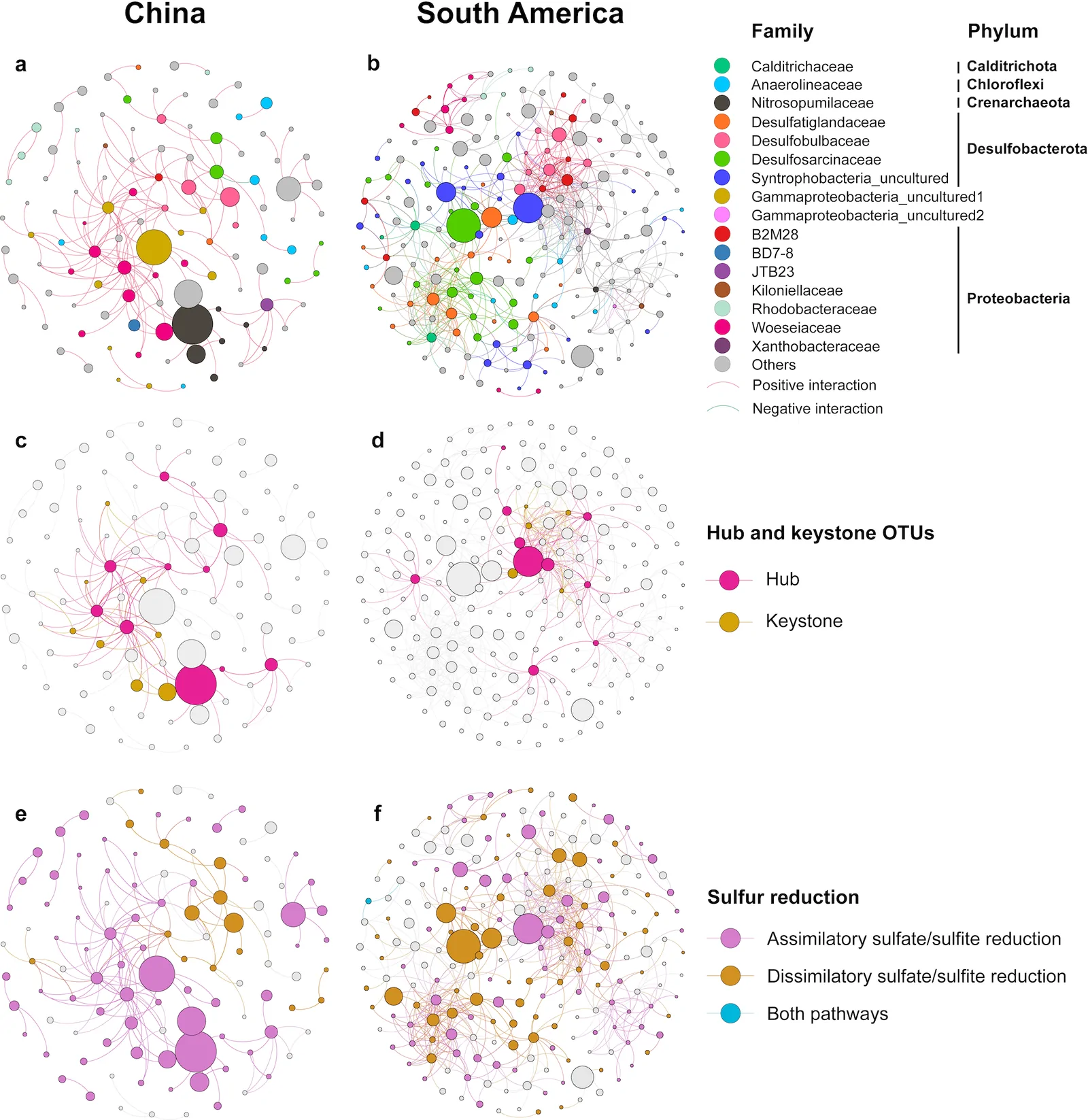
Network of co-occurring OTUs in Chinese (**a, c, and e**) and South American (**b, d, and f**) samples based on correlation analysis. Nodes of networks are colored according to family (**a and b**), role in the network (**c and d**), and the type of sulfur reduction process harbored by OTUs (**e and f**). A connection stands for a strong (*r* > 0.6) and significant (*P* < 0.05) correlation. Node size is proportional to the relative abundance of each OTU.

Next, the potential interaction between species has been identified using the co-occurrence network analysis. The co-occurrence network of the Chinese mangrove data sets consisted of 110 nodes and 174 edges, most of which were positive correlations (Fig. 4a; Dataset S1, sheet5). This network had a diameter of 11, an average number of neighbors of 3.164 (average degree), and a clustering coefficient of 0.553. Eight keystone OTUs, which are considered to possess a disproportionately detrimental effect on communities upon their removal (39), were identified in this network. Among them, there were one OTU in *Alphaproteobacteria* (*Kiloniellaceae* family) and seven OTUs in *Gammaproteobacteria,* four of which were in the *Woeseiaceae* family (Fig. 4c; Dataset S1, sheet6), indicating the indispensability of *Alpha- and Gamma-proteobacteria* for the Chinese mangrove ecosystems. The hub taxa, which are important for the community structure stability (40), included ten OTUs from *Gammaproteobacteria* (four OTUs), *Desulfobacterota* (two OTUs in *Desulfobulbaceae*, one in *Desulfosarcinaceae,* and one in *Syntrophobacterales* order), and *Nitrosopumilaceae* in *Crenarchaeota* (two OTUs) (Fig. 4c; Dataset S1, sheet6), revealing the importance of these taxa for the stability of the Chinese mangrove prokaryotic communities. In addition, correlation analyses showed that the abundance of seven keystone OTUs and five hub OTUs had a consistent relationship with the spatial and environmental parameters (negative with latitude and longitude, and positive with MAP and MDTR) (Fig. S4), suggesting that low latitude and longitude, and high MAP and MDTR were conducive to the stability of the prokaryotic communities in Chinese mangrove ecosystems. On the contrary, the abundance of two hub OTUs in *Desulfobacterota* (AY771960.1.1520 and AB602505.1.1522) was positively related to latitude and longitude, implying that *Desulfobacterota* may play a key role in maintaining the stability of the prokaryotic communities in the Chinese mangrove sites with high latitude and longitude.

The co-occurrence network of South American mangrove sediment microbiomes contained 216 nodes and 629 edges (Fig. 4b; Dataset S1, sheet5). Most of the edges in this network were with positive interactions just as in the network of the Chinese samples. The network of South American mangrove sediment microbiomes had a diameter of 12, an average degree of 5.824, and a clustering coefficient of 0.52, indicating closer linkages among microbial communities in South American mangroves than in Chinese mangrove sediments. For this network, five keystone OTUs have been identified, which belong to the *Desulfobulbaceae* family of *Desulfobacterota* (two OTUs), *Anaerolineaceae* family of *Chloroflexi* (one OTU), *B2M28* family of *Gammaproteobacteria* (one OTU), and *MBNT15* (one OTU), while the ten identified hub OTUs were of the taxa of *Desulfobacterota* (two OTUs of the *Desulfobulbaceae* family, one OTU of the *Desulfatiglandaceae* family, one OTU of the *Desulfosarcinaceae* family, and one of the *Syntrophobacterales* order), *Alphaproteobacteria* (one OTU of the *Xanthobacteraceae* family and one of the *Rhodobacteraceae* family), *Nitrosopumilaceae* of *Crenarchaeota* (one OTU), *Calditrichota* (one OTU of the *Calditrichaceae* family), and *MBNT15* (one OTU) (Fig. 4d; Dataset S1, sheet6). Correlation analyses showed that the abundances of these OTUs did not possess significant correlations with the spatial and environmental parameters, but only two OTUs seemed to relate to longitude (Fig. S4). According to this result, the stability of the microbial community in South American mangrove sediments may be less influenced by the environmental parameters. Moreover, we observed that the OTUs belonging to *Desulfobulbaceae*, *Desulfosarcinaceae,* and *Nitrosopumilaceae* (AY771960.1.1520, AB602505.1.1522, and HM171827.1.914) were the hub taxa for both Chinese and South American mangrove microbiomes. The importance of these taxa in maintaining microbial community stability in mangrove ecosystems was highlighted according to this result, and we can even infer that they may be indispensable for global mangrove ecosystems.

To explore the functions of key microbes in mangrove ecosystems, we used PICRUSt2 (41) to estimate the potential function of OTUs. Functions related to carbon fixation, nitrogen metabolisms, and sulfur metabolisms were summarized for each OTU and mapped to networks (Fig. 4e and f; Fig. S5). The results showed that 18.2% of the OTUs in the co-occurrence network for Chinese mangroves harbored the genes related to the Calvin-Benson-Bassham (CBB) pathway, while 10.9% of the OTUs contained the genes related to the Wood-Ljungdahl (WL) pathway (Dataset S1, sheet6). The similar proportion of OTUs (~30%) in the South American network contained the genes correlated with CBB and WL pathways. However, more OTUs harbored the genes related to the WL pathway (20.4% and 12.5% of OTUs for the WL and CBB pathways, respectively). For the pathways related to the nitrogen cycle, dissimilatory nitrite reduction was the most popular pathway (23.6% and 23.1% for China and South America, respectively), followed by nitrogen fixation (6.4% and 12.5% for China and South America, respectively) (Dataset S1, sheet6).

We noted that sulfur pathways were more prevalent than carbon fixation and nitrogen cycles. More than half of OTUs in the Chinese network (60.0%) and more than 30% of OTUs in the South American network (34.3%) had potential for assimilatory sulfate reduction (ASR) (Fig. 4e and f; Dataset S1, sheet7). However, only 13.6% of OTUs in the Chinese mangrove network had potential for dissimilatory sulfate reduction (DSR), while the proportion of OTUs with the same potential in the South American network reached 28.7%. In addition, it should be highlighted that 17 out of the 18 hub and keystone OTUs for the Chinese mangrove, and 7 out of the 15 hub and keystone OTUs for the South American mangrove possessed the potential for sulfate reduction (Fig. 4c through f). All these results revealed that sulfate-reducing microbes are important functional guilds for mangrove ecosystems.

### Tmap network analysis of sediment prokaryotic communities in mangrove ecosystems

**Fig 5:**
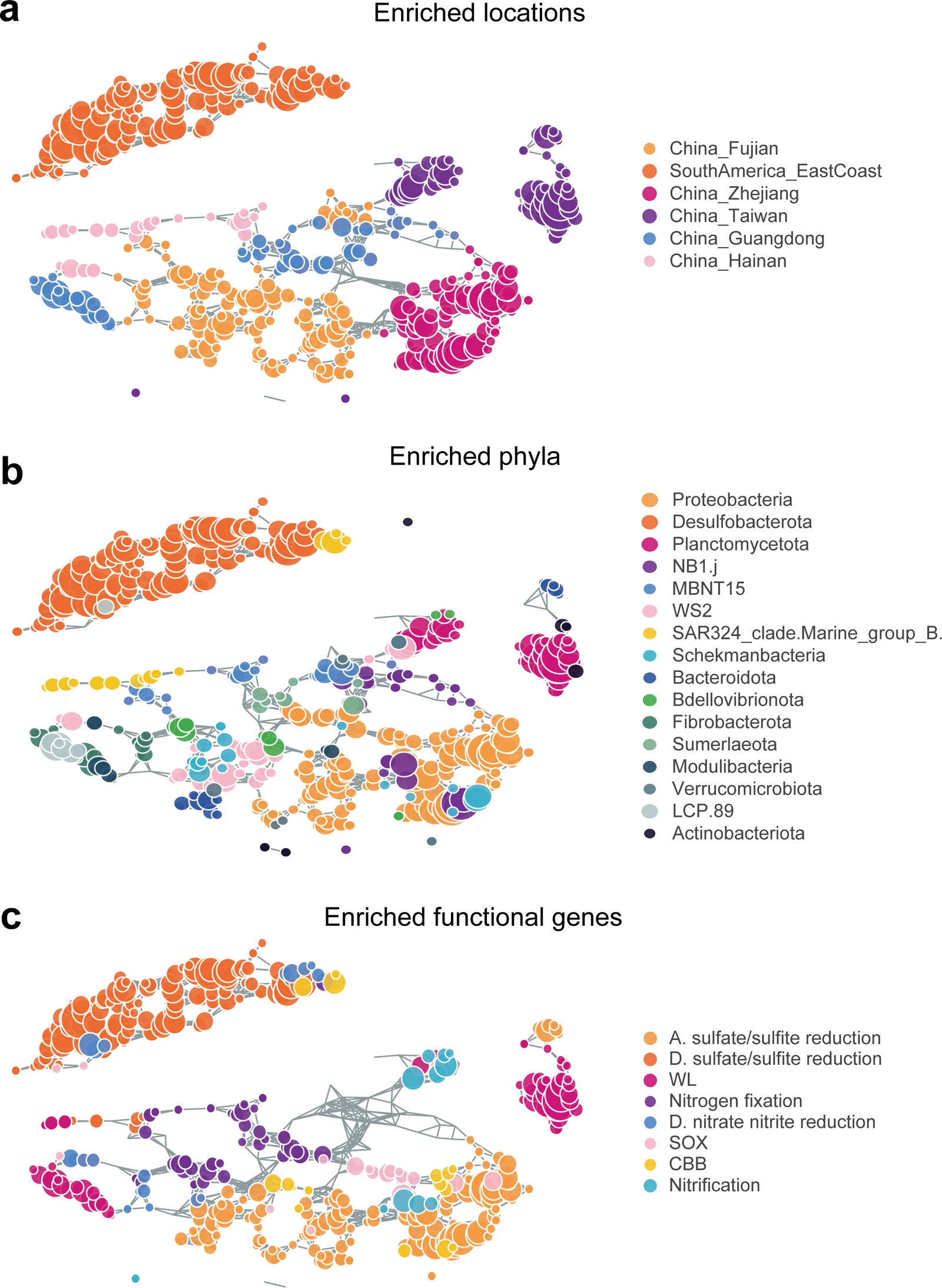
Stratification of the Chinese and South American mangrove microbiomes in the TDA network based on three groups of enriched metadata or features using tmap analysis: locations (**a**), phyla (**b**), and functional genes (**c**). Each node was colored according to its most enriched location/phyla/functional gene. Unenriched nodes were not displayed in the TDA network. A., assimilatory; D., dissimilatory; WL, Wood-Ljungdahl; SOX, sulfur oxidation; CBB, Calvin-Benson-Bassham.

Tmap analysis was conducted in all Chinese and South American samples to further identify the association of the microbial communities with sample metadata and their driver taxa (32). We observed that the topological data analysis (TDA) network was divided into several parts according to their enriched locations (Fig. 5a), exhibiting a strong association between microbiome compositions and sample locations. The subnetwork tightly associated with South America was totally isolated and away from the subnetwork enriched with Chinese samples. Moreover, the distribution of the enriched locations in the network was roughly coincident with the geographic distance. For example, either geographically or in the TDA network, Fujian was surrounded by Zhejiang, Taiwan, and Guangdong, while Hainan was adjacent to Guangdong (Fig. S1; Fig. 5a). These results further confirmed the distance-decay patterns of the microbiomes in mangrove sediments in a visualized way showing that the microbiome assembly was tightly related to locations and that the dissimilarities among the microbiomes increased with distance.

According to taxonomic compositions, the abundant phyla in mangrove ecosystems were prevalently similar. However, the phyla strongly associated with different mangrove ecosystems were divergent according to the tmap analysis of locations and prokaryotic phyla (Fig. 5a and b). The abundant phylum *Proteobacteria* was mainly dominant in Zhejiang and part of Fujian mangrove sediments, while *Planctomycetota* was associated with the microbiomes in the mangrove ecosystems in Taiwan. *Desulfobacterota* was specifically enriched in South American mangrove ecosystems but was barely enriched in China. Surprisingly, we also observed, from the tmap results (Fig. 5b), that some phyla with relatively low abundances in all samples (Dataset S1, sheet4), such as *Schekmanbacteria*, *Bdellovibrionota,* or *Fibrobacterota*, were enriched in quite a few nodes in the network, implying their specific associations with the corresponding microbiomes, which may be worth being further studied.

Combining with functional genes, the tmap analysis also obtained some specific associations. For instance, the WL pathway seemed to be enriched in Taiwan and Guangdong, while the CBB pathway was dominant in Zhejiang and specifically gathered in part of the South American samples (Fig. 5c). For the pathways related to the nitrogen cycle, dissimilatory nitrite reduction was the most popular pathway (23.6% and 23.1% for China and South America, respectively), followed by nitrogen fixation (6.4% and 12.5% for China and South America, respectively) (Dataset S1, sheet7). The genes related to ASR were mainly enriched in mangrove ecosystems in China, while those related to DSR were enriched in South American samples (Fig. 5c). This result further confirmed that ASR was absolutely dominant in the microbial communities in Chinese mangrove ecosystems, while DSR had more influence in South American mangrove ecosystems.

## DISCUSSION

This study elucidated similarities and distinctions of assembly patterns and potential key taxa of prokaryotic communities in Chinese and South American mangrove ecosystems via a large-scale data analysis. The community assemblages were analyzed for all species, abundant and rare taxa were divided based on OTU abundance, and generalists and specialists were identified via the niche width. Either in China or in South America, the other four OTU groups, except specialists, followed significant distance-decay patterns. Moreover, the topological analysis showed in a more visualized way that the dissimilarity degree of the species composition strongly depended on the geographic distance. It was reported that both historical events and contemporary environmental heterogeneity could be responsible for spatial variations in microbial communities (42). Nevertheless, the strong distance-decay relationships of the communities were more likely to confirm the importance of stochastic processes since the neutral theory predicts the decrease of the community similarity with spatial distance due to dispersal limitation rather than along environmental gradients hypothesized by the niche theory (43).

Indeed, the strong impacts of the stochastic processes on the assemblage of the four OTU groups, except specialists, were obtained by the NCM analysis. Besides, we also observed that generalists possessed the highest dispersal abilities, which was not surprising because they had relatively high niche breadths and were weakly dependent on environmental factors, as previously reported (20, 22). On the contrary, abundant OTUs were greatly limited by dispersal compared to all OTUs, rare OTUs, and generalists. We found that ~40% of the abundant OTUs belonged to generalists, ~10% were specialists, and ~50% were neither generalists nor specialists.The relatively high specificity to their habitats of the 60% of the abundant OTUs which did not belong to generalists may be the main contributor to the dispersal limitation of the abundant OTUs. Apart from stochastic processes, the effect of environmental factors on community assembly was also evaluated. Among the environmental factors analyzed in this study, MAP was the most significant factor related to the assembly patterns of all groups of OTUs in both Chinese and South American mangrove ecosystems, which is in agreement with a previous report (4). The MDTR and MAT, respectively, affected the assembly of the communities in Chinese and South American mangrove ecosystems. Nevertheless, the constrained proportions of the CCA results indicated that only a small part of community variations could be explained by these environmental factors. Since the samples studied here were collected and measured separately, other environmental factors, such as pH, organic carbon content, or sulfate content, could not be collected for all samples, leading to the incomplete analysis of the impacts of environmental factors. The difficulty in collecting the physico-chemical parameters is one of the limitations hindering the global-scale study of the mangrove ecosystems.

It was noted that specialists in both Chinese and South American mangrove ecosystems were barely affected by stochastic processes or environmental factors. The abundances of specialists were unexpectedly high in several host-associated samples, including aquatic animals, kelp forests, honeybee corpus, and human-associated samples. We found that some specialist taxa have been reported to be host-associated in previously published studies. For example, in Chinese samples, the genus *Shewanella* in *Proteobacteria* with the highest number of sequences in all samples (Dataset 1, sheet8) was identified in the gut of the benthic organism (*Munida subrugosa*) (44), the mud crab (*Scylla paramamosain*) (45), and insects [*Phasmatodea* (46), *Spodoptera litura,* and *Spilosoma obliqua* (47)]. The species *Burkholderia cepacia* of *Proteobacteria* was isolated from different root samples (48 – 50). The genus *Psychrilyobacter* of the phylum *Fusobacteriota* was found in the core gut microbiota of the mud crab (*Scylla paramamosain*) (45). The genus *Acinetobacter* of *Proteobacteria* was isolated from human specimens, such as human skin or blood (51, 52). In the South American samples, the family *Legionellaceae* of *Proteobacteria* (Dataset 1, sheet8) was found to be one of the most abundant microbes in the stool of shrimp (*Litopenaeus vannamei*) (53), and also in the feces of sea cucumbers (*Apostichopus japonicus*) (54). Some species in the family of *Dermatophilaceae* of *Actinobacteriota* were isolated from the skin of chelonids (55) and the intestinal tracts of fishes (56). The genus *Prolixibacter* of *Bacteroidota* was dominant in gut samples of four sea urchin species (*Diadema antillarum*, *Echinometra lucunter*, *Tripneustes ventricosus,* and *Lytechinus variegatus*) (57). Thus, we can infer that specialists may be controlled by the interactions of other organisms in or surrounding mangrove ecosystems. Nevertheless, the specific contributions of these organisms to the assemblage of specialists should be confirmed by further studies, for example, by collecting host-associated microbiomes in benthic animal samples in mangrove ecosystems and analyzing the interactions between these microbiomes and mangrove sediment microbial communities.

Taxonomic composition analysis revealed that the distribution of abundant phyla in different mangrove sediments was almost consistent with a few dissimilarities. The top abundant bacterial and archaeal phyla simultaneously found in all sampling locations included *Proteobacteria*, *Desulfobacterota* (except in Taiwan), *Chloroflexi, Bacteroidota*, and *Crenarchaeota*, which have been reported in different mangrove sediments (4, 5, 19, 58). As the predominant family and the majority of the hub and keystone taxa, *Gammaproteobacteria* was indicated as the core taxa that would strongly affect the microbiome assembly in Chinese mangrove ecosystems. Inconsistent with the Chinese mangrove ecosystems, *Desulfobacterota* was almost as abundant as *Proteobacteria* and was specifically enriched in South American mangrove ecosystems. Since the mean soil organic carbon contents in most South American mangrove sediments were generally higher than those in Chinese mangrove sediments (59 – 61), the possible explanation may be the positive correlation of *Desulfobacterota* with the organic carbon level, which has been reported in previous studies (62, 63). Moreover, *Desulfobacterota* was the phylum occupied by the maximum number of keystone and hub OTUs, further revealing their more important role in South American mangrove sediments.

Apart from the two dominant phyla, several other taxa may also prominently affect the community assembly and stability. The archaeal hub OTUs belonging to *Nitrososphaeria* (a class characterized as ammonia-oxidizing archaea) (64) were found in both Chinese and South American mangrove ecosystems (Dataset S1, sheet6), implying their importance in the stability of the community structure in mangrove ecosystems in both locations. Apart from *Proteobacteria*, *Desulfobacterota,* and *Crenarchaeota*, in South American mangrove ecosystems, the keystone and hub OTUs contained the other three taxa. *Anaerolineae* of *Chloroflexi*, which has been identified as a dominant keystone taxon in Yunxiao mangrove sediments (5), is a prevalent anaerobic fermenter (65, 66). *Calditrichota* is an anaerobic organoheterotroph and is abundant in marine sediments (67, 68). *MBNT15* is distantly related to *Desulfobacterota* and is capable of degrading complex polymers (69). In addition to these relatively abundant taxa, some rare taxa, for example, *Schekmanbacteria*, which is supposed to contain sulfate-/sulfite-reducing pathways (70), and *Fibrobacterota*, which is thought with the function of cellulose hydrolysis (71), gathered in certain mangrove sediments in China according to the tmap analysis. These results implied that these rare taxa may be specifically associated with the microbiome assembly in part of the mangrove sediments, and their contribution should not be neglected.

In terms of metabolic functions, sulfate reduction was the most prevalent in mangrove ecosystems compared to other functions analyzed in this study. In the co-occurrence network, OTUs harboring genes related to the pathway of sulfate reduction were much more than OTUs with the potential of carbon fixation or nitrogen metabolisms (Fig. 4e and f; Fig. S5). According to the enrichment analysis using tmap, sulfate reduction was the most enriched in more than half of the nodes (Fig. 5c). This is not surprising because mangrove sediments are generally considered anaerobic and rich in sulfate and organic carbon, providing appropriate circumstances for sulfate-reducing prokaryotes (72, 73). Apart from sulfate cycling, some sulfate-reducing prokaryotes can also promote diverse metabolisms, such as carbon (74, 75), nitrogen (76, 77), and metal cycling (78, 79), revealing their crucial roles as biogeochemical linkers. Although sulfate-reducing prokaryotes were observed to be dominant in both Chinese and South American mangrove sediments, we noted that compared to DSR, ASR, which transforms sulfate to organic sulfur (80), was more prominent in Chinese mangrove ecosystems, while DSR, a pathway tightly associated with organic carbon mineralization in anoxic environments (81, 82), played a more important role in South American mangrove sediments. However, the prediction of functional genes based on 16S rRNA was rough. Further analysis of metagenomic/metatranscriptomic data and chemical composition of sediments should be performed to untangle the key roles of sulfate reduction and the factors leading to distinctions in a dominant mode in Chinese and South American mangroves.

The results obtained with the Chinese and South American mangrove sediment samples revealed that, although distant, the mangrove ecosystems generally display analogous features in terms of community assembly patterns and functioning. Nevertheless, a global-scale analysis is indispensable for comprehensively exploring the assembly patterns of microbial communities in mangrove ecosystems and the governing factors. To our knowledge, the studies investigating and comparing the global-scale mangrove microbiomes are still lacking. This is mainly due to the diverse primers used to sequence the 16S rRNA data for different mangrove sediment samples, which would induce non-negligible bias for community-level analysis, as universally acknowledged and further proved in our study (Supplemental file 1). For example, in addition to the data analyzed in this study, some Indian mangrove sediment samples were sequenced with 341F-805R (83, 84), while the 27F-519R primer was used for Australian mangrove sediment sample sequencing (85). Thus, a standardized protocol for sequencing 16S rRNA gene amplicons of mangrove sediment microbiomes with a unified primer should be constructed to realize the global-scale analysis. Moreover, a standardized protocol can facilitate the comparison of mangrove microbiomes with reference data sets (e.g., EMP) in other biomes.

### Conclusions

Although distant, analogous features in community assembly patterns and key functional guilds were explored in Chinese and South American mangrove ecosystems. Specifically, the assemblages of the entire communities, abundant taxa, rare taxa, and generalists were strongly driven by stochastic processes with significant distance-decay patterns and were partly affected by environmental factors. However, specialists were mainly affected by the behaviors of other organisms in or surrounding mangrove ecosystems, which should be further confirmed via investigating related host-associated samples. The generally dominant phyla in both Chinese and South American mangrove ecosystems were *Proteobacteria*, *Desulfobacterota*, *Chloroflexi*, *Bacteroidota,* and *Crenarchaeota*, in which some taxa (mainly *Gammaproteobacteria*, *Desulfobulbia*, or *Nitrososphaeria*) acted as putative keystones or hubs, playing important roles in maintaining community structures. Nevertheless, the contribution of these taxa to microbial communities in different mangrove ecosystems exhibited dissimilarities. Moreover, our study underlined the dominance of sulfate-reducing prokaryotes in both Chinese and South American mangrove ecosystems. These important functional taxa, to which more than 70% of the keystones and hubs belonged, may make great contributions to maintaining the linkage and stability of the communities. Thus, it would be worth making more efforts in further study to explore in depth how sulfate-reducing prokaryotes maintain community structures and drive element transformation in mangrove ecosystems. Finally, to facilitate the global-scale analysis of mangrove microbiomes and the comparison with the reference data sets in other biomes, we are making an appeal to the Mangrove Microbiome Initiative to standardize the protocol for sequencing mangrove sediment 16S rRNA data with a unified primer.

## MATERIALS AND METHODS

### Sample collection and metadata measurement

In this study, a total of 380 samples from Chinese and South American mangroves were analyzed. Among them, 217 samples of the 0–20 cm sediment were collected from 12 representative mangrove nature reserves in four provinces in south-eastern China, including Zhejiang (65 samples), Fujian (104 samples), Guangdong (24 samples), and Hainan provinces (24 samples) (Fig. S1; Dataset S1, sheet1). The data from Taiwan province (62 samples of the 0–30 cm sediment) (37) and eight mangroves in South America (101 samples of the 0–20 cm or surface sediment) (29, 38) were retrieved from NCBI databases (Fig. S1; Dataset S1, sheet1). The latitudes and longitudes of the sampling sites were recorded using a GPS unit. MAT, MAP, and MDTR were calculated based on the data from the National Oceanic and Atmospheric Administration (NOAA) (Dataset S1, sheet1).

### 16S rRNA gene amplicon sequencing

Sediment genomic DNA was extracted from the sediment samples using a DNeasy PowerSoil kit (Qiagen, Germany) according to the manufacturer’s instructions. Triplicate DNA extracts were pooled for each sample and stored at −20°C for further use. The DNA samples were amplified using 515F-806R primer pairs. Subsequent library preparation and sequencing processes followed two pipelines (the correspondence of the sample and pipeline was marked in Dataset S1, sheet1):

1. Sequencing libraries were generated using a NEBNext Ultra II DNA Library Prep Kit for Illumina (New England Biolabs, MA, USA) following the manufacturer’s recommendations, and index codes were added. The library quality was assessed on a Qubit@ 2.0 Fluorometer (Thermo Fisher Scientific, MA, USA). At last, the library was sequenced on the Illumina MiSeq platform, and 250-bp paired-end reads were generated.
2. Sequencing libraries were generated using a Nextflex rapid DNA-Seq kit (BIOO SCIENTIFIC, TX, USA) following the manufacturer’s recommendations. The library quality was assessed on a Qubit@ 2.0 Fluorometer (Thermo Fisher Scientific, MA, USA). At last, the library was sequenced on the Illumina MiSeq platform, and 300-bp paired-end reads were generated.

### Data processing

Raw data sequenced with 515F-806R primer pairs were combined with the reference data from Taiwan (sequenced by 515F-806R) to form the China data set (279 samples). The reference data from South America (sequenced by 515F-806R) were combined to form the South America data set (101 samples).

All data sets were first filtered to remove low-quality sequences using Sickle with a threshold of 20 for trimming based on the average quality in a window and a threshold of 20 to keep a read based on the length after trimming (step for data cleaning) (86). Then, the recommended QIIME pipeline of the Earth Microbiome Project (EMP) (87) was applied to analyze the data sets. Briefly, all sequences were merged using the join_paired_ends.py script. After the removal of chimeras using ChimeraSlayer (step for data decontamination) (88), the remaining sequences were clustered by a closed-reference OTU picking process at 97% sequence identity with the SILVA123 database (denoizing) (89). Representative sequences of OTUs were then aligned to the SILVA138.1 database to obtain taxonomic information. All OTU tables were rarefied to the same number (10,000 sequences per sample), and all samples with less than 10,000 sequences were excluded. Finally, 279 China samples and 101 South American samples were included for the following analysis. For comparative analyses with the EMP data, the two data sets were merged with emp_cr_silva_16S_123.release1.biom using QIIME (90).

### Statistical analysis

#### Identification of abundant and rare OTUs, generalists, and specialists

In this study, abundant OTUs were defined as OTUs with a relative abundance of >1% in at least one sample, while rare OTUs were OTUs with a relative abundance of <1% in all samples (91, 92). Generalists and specialists were defined according to Liao et al. (22). Generalists were OTUs enriched in a wide range of environments (larger niche width indexes), and the enrichment of specialists occurred in narrow environments (smaller niche width indexes). The niche width index of each OTU was calculated using EcolUtils packages (93) in R. OTUs within the top 10% of the niche width index were defined as generalists, and those at the bottom 10% were regarded as specialists. It should be noted that all OTUs with the minimum niche width index were regarded as specialists, even if their proportion was higher than 10%.

#### Microbial community assembly patterns

Mechanisms of community assembly include both niche theory and neutral theory. To calculate the distance-decay of microbial communities in mangrove ecosystems, dissimilarity indices of each sample pair were calculated using the vegan package (94) in R software. Then, a linear regression was fitted to test the correlation between the community similarity and spatial distance of each sample pair. To evaluate the role of stochastic processes in microbial community assembly in mangrove ecosystems, the neutral community model (NCM) (95) was used. The non-linear least squares was used to fit the relationship between occurrence frequency and relative abundance of OTUs using minpack.lm package (96) in R software. To explore the effects of environmental factors on microbial communities, the correlation between environmental factors and the relative abundance of OTUs was calculated using the vegan package (94). First, detrended correspondence analysis (DCA) was performed for all microbial data sets with decorana function. As the lengths of all the first axes were >4, canonical correspondence analysis (CCA) was performed to calculate the correlation. A CCA-based variation partition analysis (VPA) was performed to determine the relative proportions of community variations explained by the environmental factors. Besides, we combined mangrove OTU table and EMP OTU table of 27,751 samples in diverse ecosystems and then estimated the influence of microbiota from other environments on specialists using Sourcetracker2 (97). Furthermore, the abundance of the specialist OTUs in other environments was calculated.

### Network analysis

#### OTU-level interactions and key taxa via co-occurrence network analysis

The co-occurrence network of prokaryotes based on the OTU table was performed using SparCC (98). Only the OTUs with a relative abundance of >0.1% in at least one sample were included in the network analyses (99, 100). Then, the network was screened using R software by keeping the relations with *P* < 0.05 and *r* > 0.6. To identify the hub OTUs and keystone OTUs, the values of betweenness, centrality, closeness, and mean degree were calculated using the igraph package (101). Hub OTUs were defined as OTUs occupying key positions in ecological networks and being essential for the stability of the community structure (the nodes with top 30% degree centrality and top 30% betweenness) (40). Keystone OTUs were considered to possess a disproportionate detrimental effect on the community upon their removal (the nodes with top 30% mean degree, top 30% closeness, and last 30% betweenness) (39). To explore the metabolic potentials of the microbiota in mangrove sediments, PICRUSt2 analyses (41) were employed to predict potential metabolisms related to carbon fixation, nitrogen metabolisms, and sulfur metabolisms.

#### Associations of taxonomic features with sampling locations using tmap framework

The mapper algorithm in the tmap framework (32) was adopted to transform the OTU table of all the 380 sediment samples sequenced with 515F-806R to a TDA network. In the TDA network, the profiles of these samples were projected from high-dimensional data points into a low-dimensional space using MDS filter. Then, the samples were clustered into different nodes and linked with each other depending on their topological properties with a set of parameters defined according to the data size and microbiome variation (resolution = 40, overlap = 0.75, mini-sample = 2, and percentile eps threshold = 98th). After the construction of the TDA network, the values of metadata/microbiome features, such as the sample location or the abundance of potential functional genes in each sample, were mapped to the network. Then, the enrichment level of a given variable (one of the metadata or microbiome features) on each node in the network was quantified and expressed as the spatial analysis of functional enrichment (SAFE) score, which reflects the relative association extent of the node with a variable. Besides, the SAFE score can also help characterize the interrelations among distinct variables via the co-enrichment patterns of these variables.

## Data Availability

All raw sequences from the current study have been deposited in NODE under the accession numbers OEP001343, OEP001474, OEP001667, OEP001673, OEP001828, OEP001935 to OEP001948, OEP002010, and OEP002621.

## SUPPLEMENTAL MATERIAL

Dataset S1 sheet1 to sheet8 spectrum.01577-23-s0001.xlsx Supplemental tables and captions at the end of main text.

Fig. S1 to S5, Supplemental file 1 spectrum.01577-23-s0002.docx Supplemental figures and supplemental file for comparing the impact of different primer pairs on 16S rRNA sequencing results.

ASM does not own the copyrights to Supplemental Material that may be linked to, or accessed through, an article. The authors have granted ASM a non-exclusive, world-wide license to publish the Supplemental Material files. Please contact the corresponding author directly for reuse.
