# Supplementary material for: Analogous assembly mechanisms and functional guilds govern prokaryotic communities in mangrove ecosystems of China and South America
Source: Microbiol Spectr. 2023 Sep 5;11(5):e01577-23. doi: 10.1128/spectrum.01577-23 (PMC10580968; doi:10.1128/spectrum.01577-23)
Supplement: Fig. S1 to S5, Supplemental file 1 — Supplemental figures and supplemental file for comparing the impact of different primer pairs on 16S rRNA sequencing results. [file spectrum.01577-23-s0002.docx]

**Supplemental figures**

**
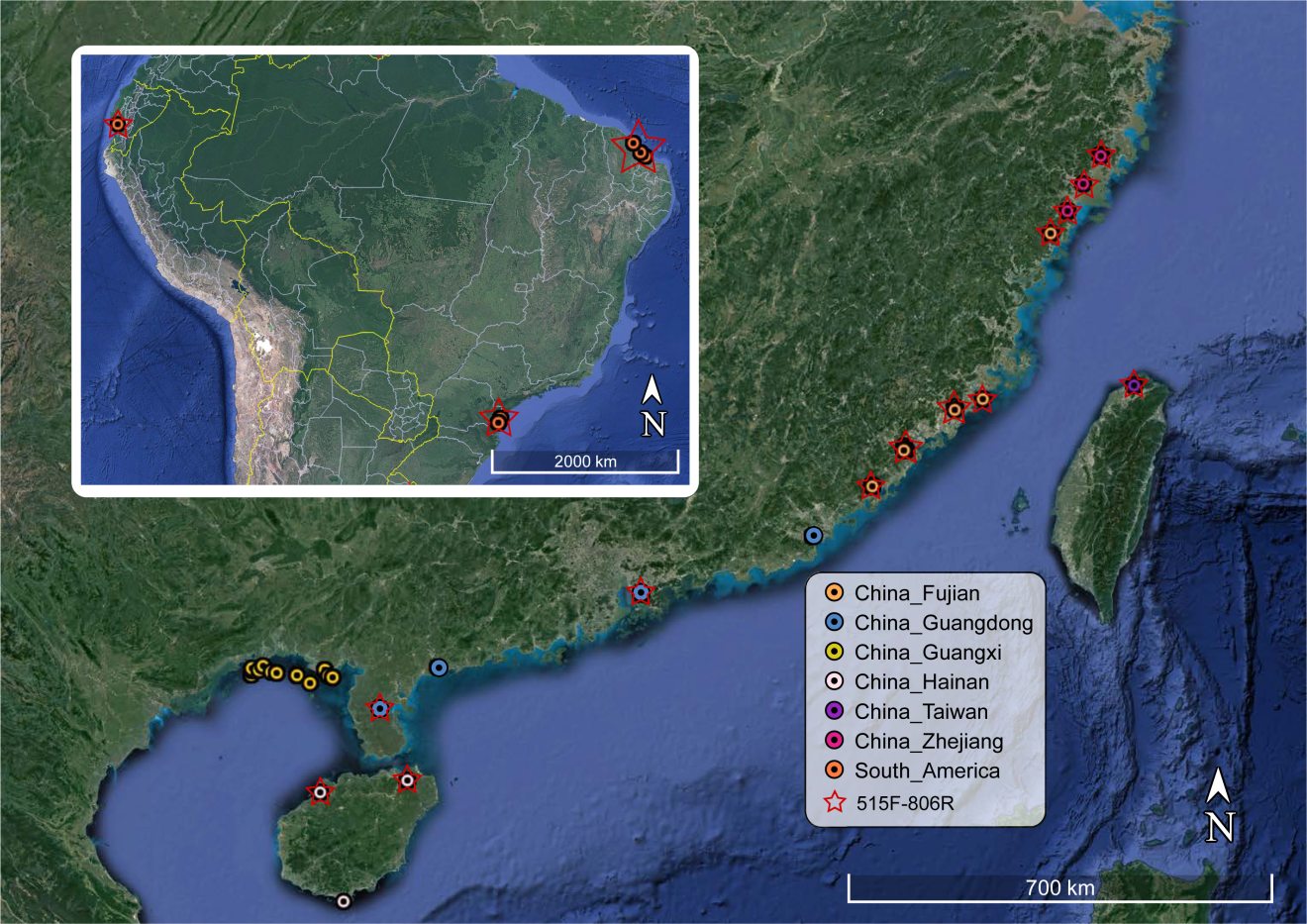
**

Fig. S1 Sampling sites of the mangrove sediments.


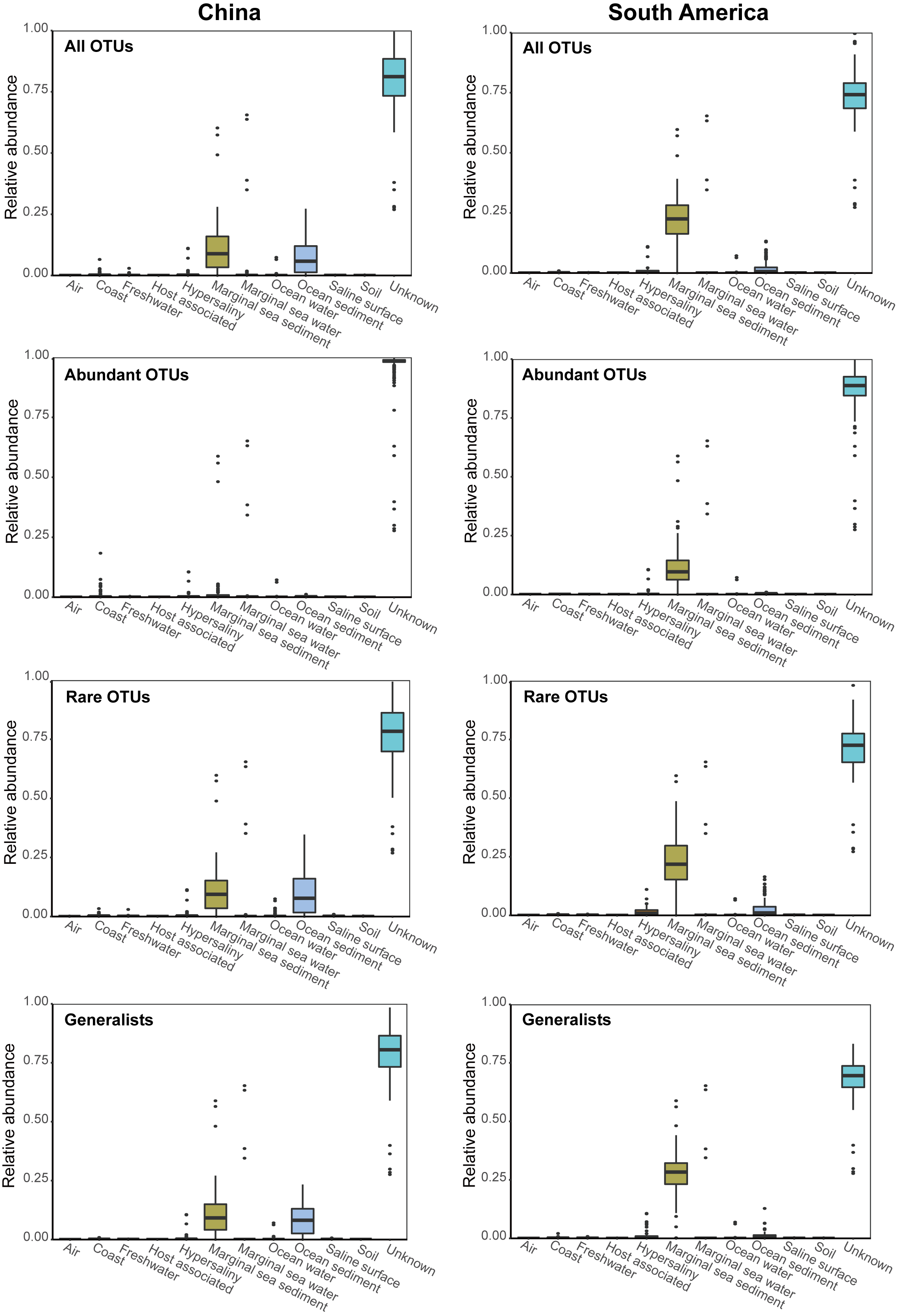


Fig. S2 Boxplot of the relative abundances of all OTUs, abundant OTUs, rare OTUs and generalists identified in Chinese and South American mangrove sediments in other biomes.


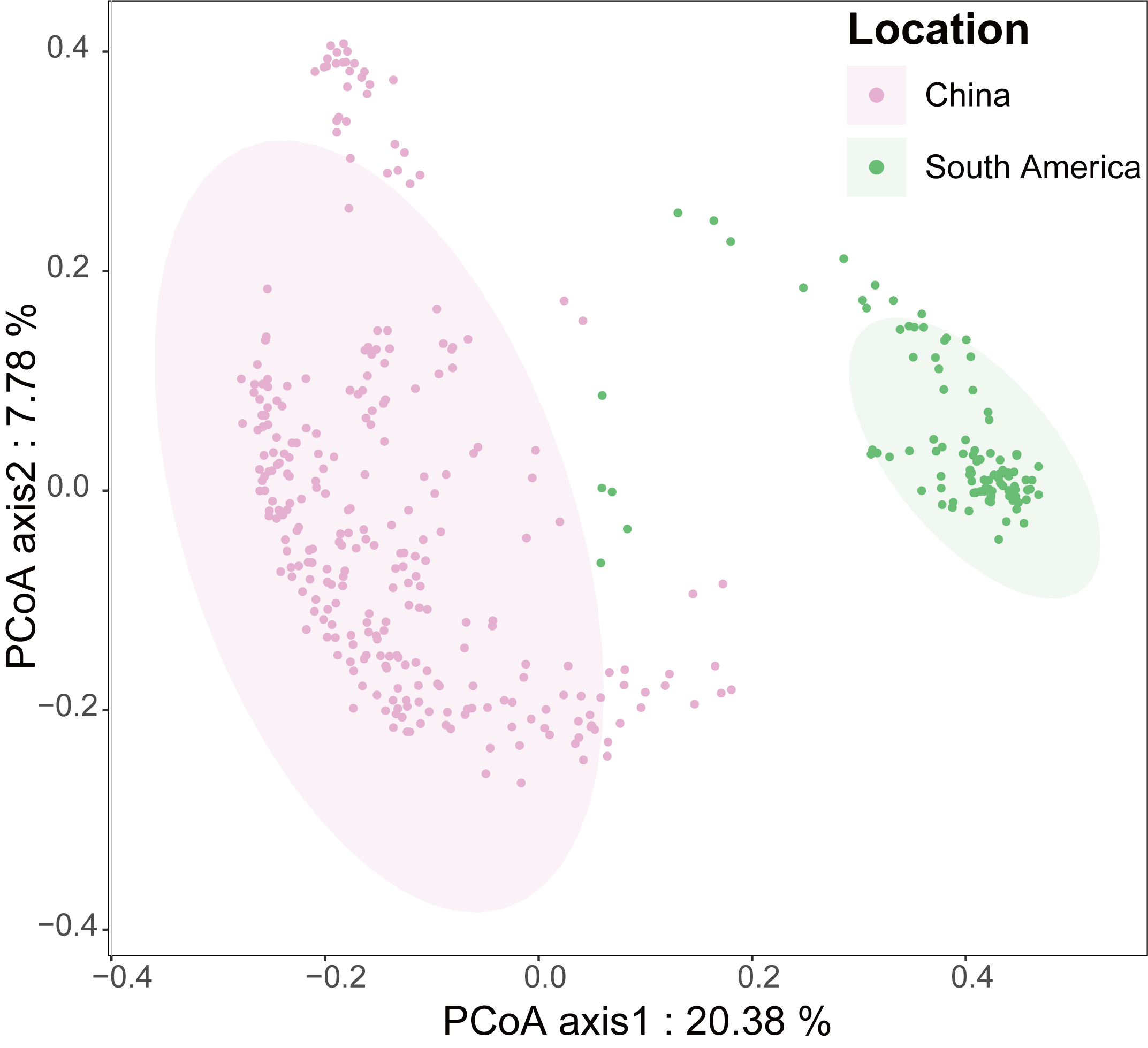


Fig. S3 Principal coordinates analysis (PCoA) plot of microbial community compositions in Chinese and South American mangrove sediments.

**
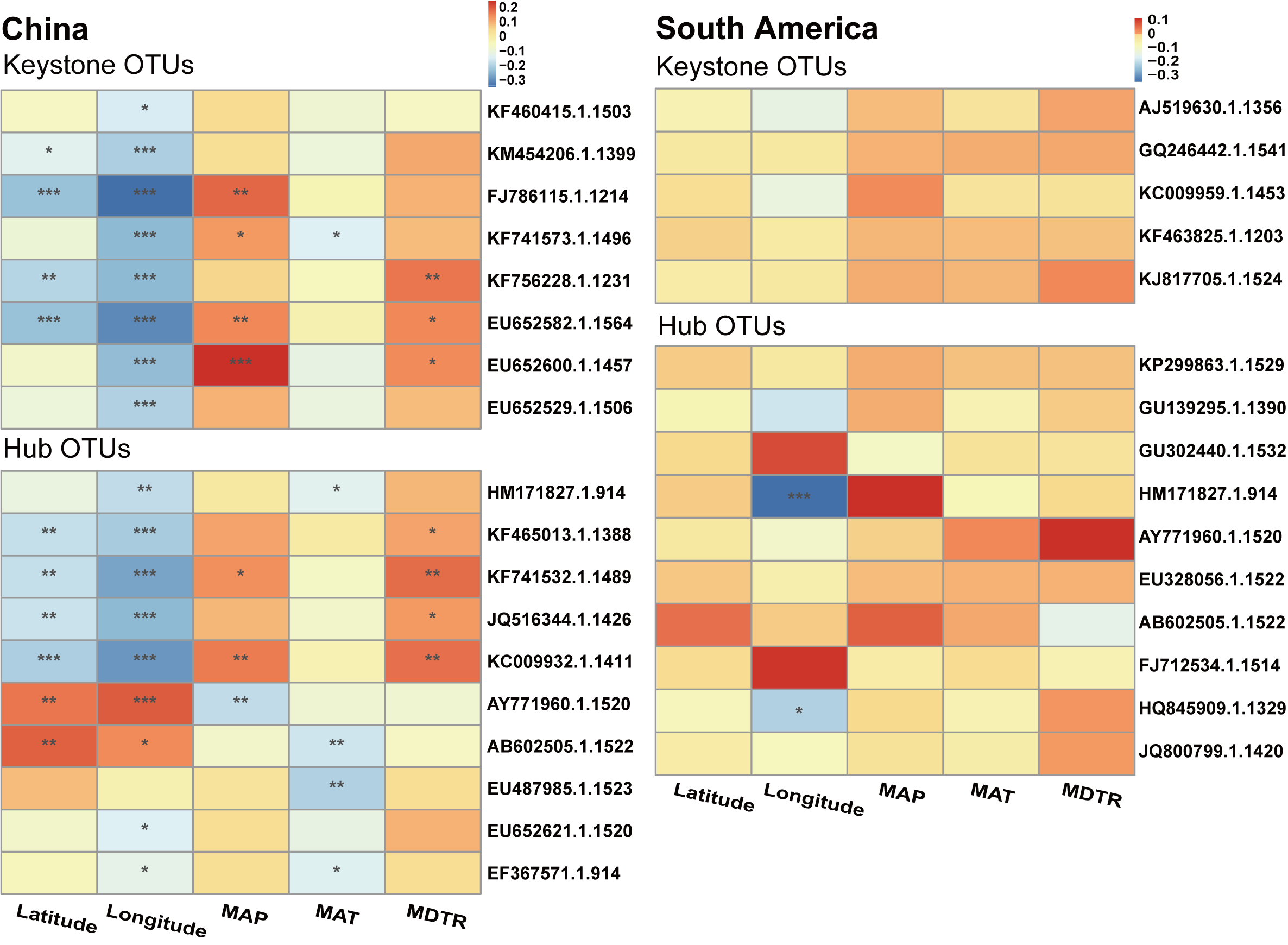
**

Fig. S4 Correlations of the keystone and hub OTUs of the Chinese and the South American mangrove ecosystems with environmental factors.

**
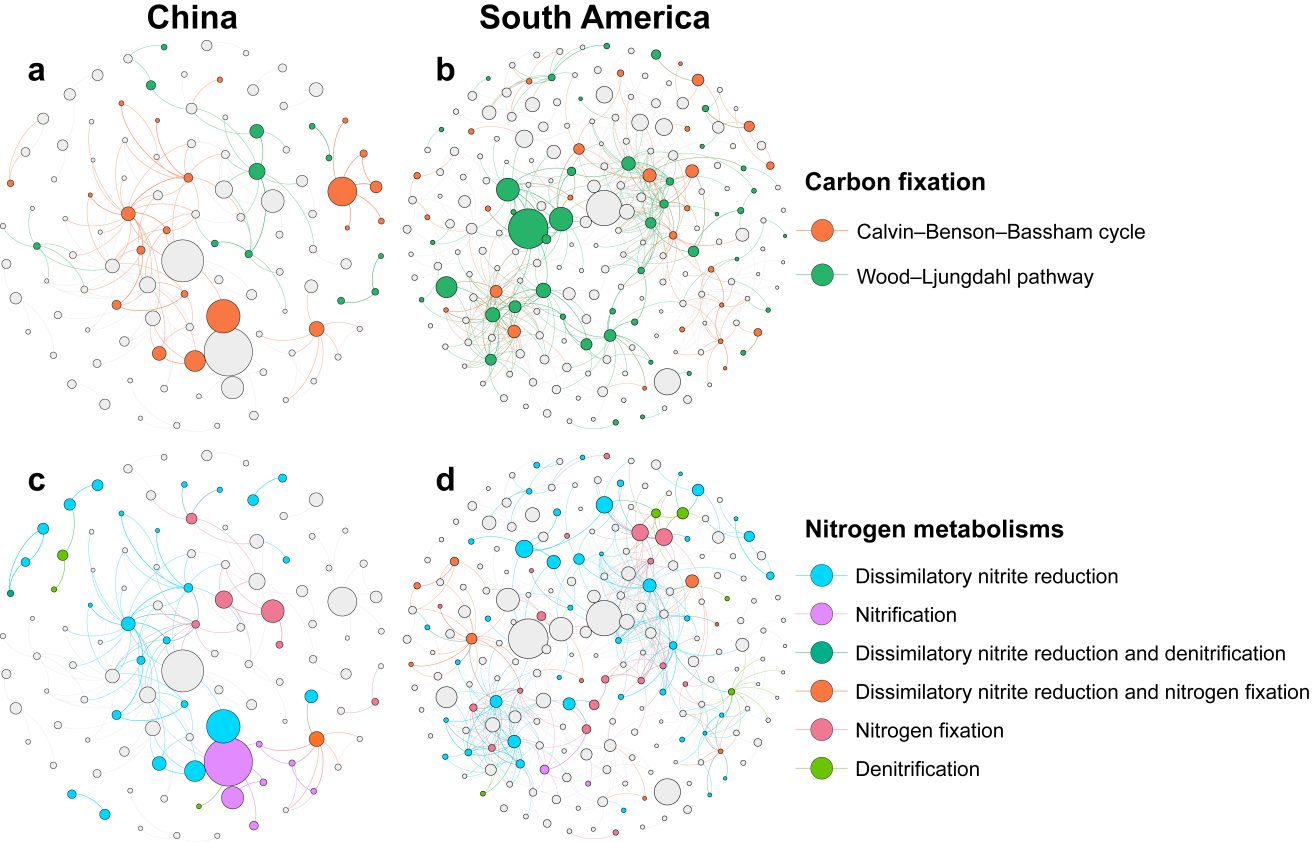
**

Fig. S5 Carbon fixation and nitrogen metabolisms harbored by the co-occurring OTUs respectively in Chinese and South American mangrove ecosystems.

**Supplemental file 1. The effects of inducing data sequenced with different primers on mangrove microbial community study**

Due to the preferences of different instruments, protocols, sample types, customs, projects and institutes, there are many environmental microbial datasets of 16S rRNA data sequenced using different primers in the public databases. As it is a consensus that using different primers will cause unavoidable bias, many reference datasets could not be included in the analyses. We wondered if it is possible that some primers may induce little bias that may not affect some parts of the final conclusions. To explore the effects of different primers, the 16S rRNA datasets of the Chinese mangrove sediments sequenced using four pairs of primers (338F-806R, 341F-806R, 515F-806R, and 515F-907R) were introduced and analyzed.

**Methods**

For analyzing the impacts of the 16S rRNA results sequenced with different primers on the community-level analysis, a total of 706 samples (including the 279 samples collected in China and in Taiwan province) from 17 mangroves in China were collected (Fig.S1; Dataset S1, sheet1). The sample DNA (except the Taiwan data that were downloaded from public databases) was extracted and sequenced with 4 primer pairs (338F-806R, 341F-806R, 515F-806R, and 515F-907R) following five pipelines (the correspondence of the sample, primer and pipeline was marked in Dataset S1, sheet1):

1. Sediment genomic DNA was extracted from the sediment samples using DNeasy PowerSoil kit (Qiagen, Germany) according to the manufacturer’s instructions. Triplicate DNA extracts were pooled for each sample and stored at -20 °C for further use. Sample DNA was amplified using the corresponding primer pairs. Sequencing libraries were generated using NEBNext® Ultra™ II DNA Library Prep Kit for Illumina® (New England Biolabs, MA, USA) for Illumina® (New England Biolabs, MA, USA) following manufacturer's recommendations and index codes were added. The library quality was assessed on the Qubit@ 2.0 Fluorometer (Thermo Fisher Scientific, MA, USA). At last, the library was sequenced on a Illumina MiSeq platform and 250 bp paired-end reads were generated.
2. Sediment genomic DNA was extracted from the sediment samples using DNeasy PowerSoil kit (Qiagen, Germany) according to the manufacturer’s instructions. Triplicate DNA extracts were pooled for each sample and stored at -20 °C for further use. Sample DNA was amplified using the corresponding primer pairs. Sequencing libraries were generated using Nextflex rapid DNA-Seq kit (BIOO SCIENTIFIC, TX, USA) following manufacturer's recommendations. The library quality was assessed on the Qubit@ 2.0 Fluorometer (Thermo Fisher Scientific, MA, USA). At last, the library was sequenced on a Illumina MiSeq platform and 300 bp paired-end reads were generated.
3. Sediment genomic DNA was extracted from the sediment samples using a Power Soil DNA Isolation Kit (MoBio, Carlsbad, CA, USA) according to manufacturer’s instructions. Triplicate DNA extracts were pooled for each sample and stored at -20 °C for further use. Sample DNA was amplified using the corresponding primer pairs. PCR amplification was performed in a total volume of 50 μL containing 10 μL buffer, 0.2 μL Q5 high-fidelity DNA polymerase, 10 μL high GC enhancer, 1 μL dNTP, 10 μM of each primer, and 60 ng of microbial community DNA. Thermal cycling conditions were as follows: an initial denaturation at 95 °C for 5 min, followed by 15 cycles at 95 °C for 1 min, 50 °C for 1 min, and 72 °C for 1 min, with a final extension at 72 °C for 7 min. The PCR products from the first step of PCR were purified using VAHTSTM DNA Clean Beads. A second round of PCR was then performed in a 40 μL reaction containing 20 μL 2× Phusion HF MM, 8 μL ddH2O, 10 μM of each primer, and 10 μL PCR products from the first step. Thermal cycling conditions were as follows: an initial denaturation at 98 °C for 30 s, followed by 10 cycles at 98 °C for 10 s, 65 °C for 30 s, and 72 °C for 30 s, with a final extension at 72 °C for 5 min. All PCR products were quantified using Quant-iT™ dsDNA HS Reagent and were pooled. Highthroughput sequencing of 16S rRNA genes was performed using Illumina Hiseq 2500 platform and 250 bp paired-end reads were generated.
4. Sediment genomic DNA was extracted from the sediment samples using DNeasy PowerSoil kit (Qiagen, Germany) according to the manufacturer’s instructions. Triplicate DNA extracts were pooled for each sample and stored at -20 °C for further use. Sample DNA was amplified using the corresponding primer pairs. Sequencing libraries were generated using TruSeq® DNA PCR-Free Sample Preparation Kit following manufacturer's recommendations. The library quality was assessed on the Qubit@ 2.0 Fluorometer (Thermo Fisher Scientific, MA, USA). At last, the library was sequenced on a Illumina Hiseq 2500 platform and 250 bp paired-end reads were generated.
5. Sediment genomic DNA was extracted from the sediment samples using PowerSoil DNA isolation kit (MO BIO, USA) according to the manufacturer’s instructions. Triplicate DNA extracts were pooled for each sample and stored at -20 °C for further use. Sample DNA was amplified using the corresponding primer pairs. Sequencing libraries were generated using Nextflex rapid DNA-Seq kit (BIOO SCIENTIFIC, TX, USA) following manufacturer's recommendations. The library quality was assessed on the Qubit@ 2.0 Fluorometer (Thermo Fisher Scientific, MA, USA). At last, the library was sequenced on a Illumina MiSeq platform and 300 bp paired-end reads were generated.

The 16S rRNA data were processed using the same method as in the manuscript. Then, the PCoA analysis, distance-decay relationship estimation, NCM analysis, CCA analysis and co-occurrence network analysis were conducted to this dataset in the same way as in the manuscript.

**Results**

Then PCoA analysis of all samples was conducted to explore the dissimilarities of data sequenced with the four primers. The results showed that the samples using primer 515F-907R were significantly different from those with other primers (Fig. Sf1). Thus, the samples using primer 515F-907R were excluded in the following analyses.

The same analyses as for the Chinese samples sequenced with the 515F-806R primer were repeated with the three-primer data to test the bias induced by the different primers. The distance-decay results showed the similar patterns compared to the results of 515F-806R samples (Fig. 1a; Fig. Sf2a). The sharp slopes of the distance-decay relationships were observed for the communities consisting of all OTUs, the abundant OTUs, the rare OTUs, and the generalists (p<0.01, R = -0.26 ~ -0.35), while the slope for the specialists was significantly gentler. The NCM analysis also showed similar results with that of the 515F-806R samples (Fig. 1b; Fig. Sf2b). A large fraction of the community variations of all OTUs, the abundant OTUs, the rare OTUs, and the generalists (R^2^ ranged from 0.59 to 0.87) were observed, whereas the NCM of the specialists estimated almost none of the relationship between the occurrence frequency and the abundance variations (R^2^ < 0).


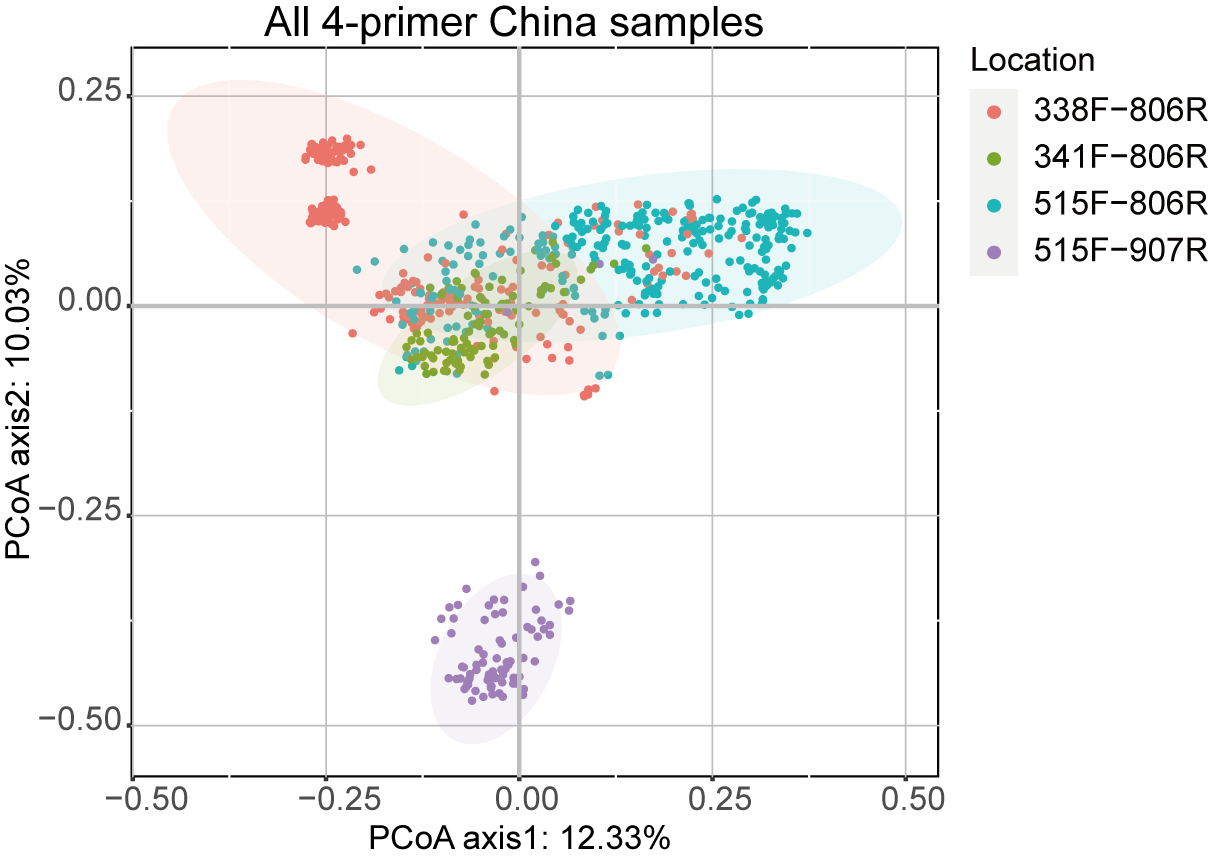


Fig. Sf1 Principal coordinates analysis (PCoA) plot of samples from Chinese mangrove sediments sequenced with 4 different primers.


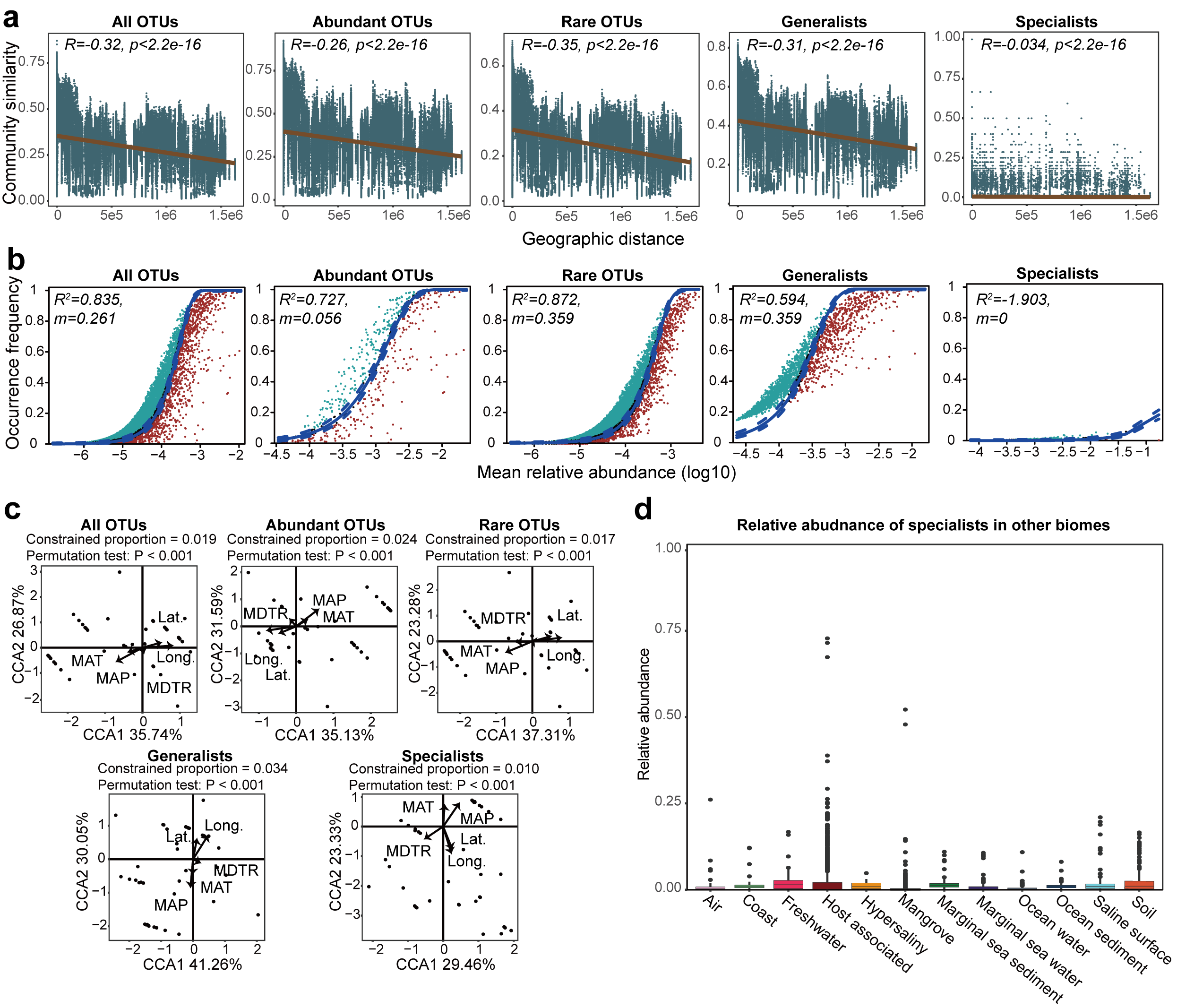


Fig. Sf2 Assembly patterns of five prokaryotic (sub)-communities with different traits respectively composed of all OTUs, abundant OTUs, rare OTUs, generalists and specialists in Chinese mangroves analyzed with the data sequenced with 3 different primers (338F-806R, 341F-806R, and 515F-806R). (a) Distance-decay relationships for the five (sub-)communities. Slope, distance-decay slope (i.e., rate at which similarity decreases with distance); p, significance of the model. (b) Fitting neutral community model (NCM) to the five (sub-)communities. Solid blue lines denote the best fit of the NCM; dashed blue lines represent 95% confidence intervals around the best-fit neutral model. R^2^, goodness of fit to the model; m, estimated immigration rate. (c) Canonical correspondence analysis (CCA) of the relationship between environmental factors and the composition of the five microbial (sub-)communities. Arrows represent quantitative environment factors, while dots represent different samples. Long., longitude; Lat., latitude; MAT, mean annual temperature; MAP, mean annual precipitation; MDTR, mean daily temperature range. (d) Boxplot of the relative abundances of the specialists in other biomes.

In addition, the CCA results suggested that only a small proportion of microbial community variations was explained by the environmental and spatial factors studied in this study (constrained proportions ranged from 0.010 to 0.034), which was the same as the results of the 515F-806R samples (Fig. 2a; Fig. Sf2c). Besides, among diverse habitats, the mean abundances of the specialists were only high in certain host-associated samples (Fig. Sf2d). Altogether, the introduction of the other two-primer datasets did not change the main conclusion about the factors affecting the assembly of microbial communities in Chinese mangrove sediments.

Further, the co-occurrence network based on the three-primer datasets were built. When the r-value cutoff was set to 0.6, the resulting network was only a small network with 10 nodes and 8 edges (Fig. Sf3a). Only if the r-value cutoff reduces to 0.5, a reasonable scale network (with 64 nodes and 129 edges) could be built (Fig. Sf3b). A possible reason is that different primers may change the relative abundance of some OTUs, which may reduce their co-occurrence significancy. While setting the r-value cutoff as 0.5, the resulting network was with a diameter of 4, a weighted degree of 4.32, and an average degree of 4.03. For this network, 9 keystone OTUs were identified, which belonged to Desulfobacterota, Firmicutes, Gemmatimonadota, Halobacteria, Alpha-, and Gamma-proteobacteria, while 10 hub OTUs were in the taxa of Bathyarchaeota, Desulfobacterota, Alpha-, and Gamma-proteobacteria (Fig. Sf3c). The results indicated that the introduction of the other primer data would change the results of the keystone and hub OTUs by involving more other taxa. We also predicted the potential metabolisms of each OTU and mapped the results to the network. The proportion of the popular metabolisms based on three-primer datasets was similar to those based on 515F-806R datasets (Fig. Sf3d-f). The CBB pathway was also the most popular carbon fixation pathway with 23.4% OTUs harboring the related genes. Dissimilatory nitrite reduction (28.1%) and nitrogen fixation (12.5%) were the two dominant pathways in the nitrogen cycle. The proportion of the OTUs possessing the potential for ASR (56.3%) was greatly higher than those with DSR (9.4%). Most of the keystone and hub OTUs (14 out of 19) were potentially capable of the sulfate reduction (Fig. Sf3c, d).

In conclusion, although the introduction of the datasets sequenced with primers 338F-806R and 341F-806R may not change the main conclusions of the community assembled mechanisms. But it would greatly influence the taxonomic compositions and hence change the co-occurrence connections of the OTUs and induce noises to the determination of the key players of the community. Thus, we should try not to involve the datasets of different primers, and carefully consider the reliability of the conclusions if we have to use multiple-primer datasets.


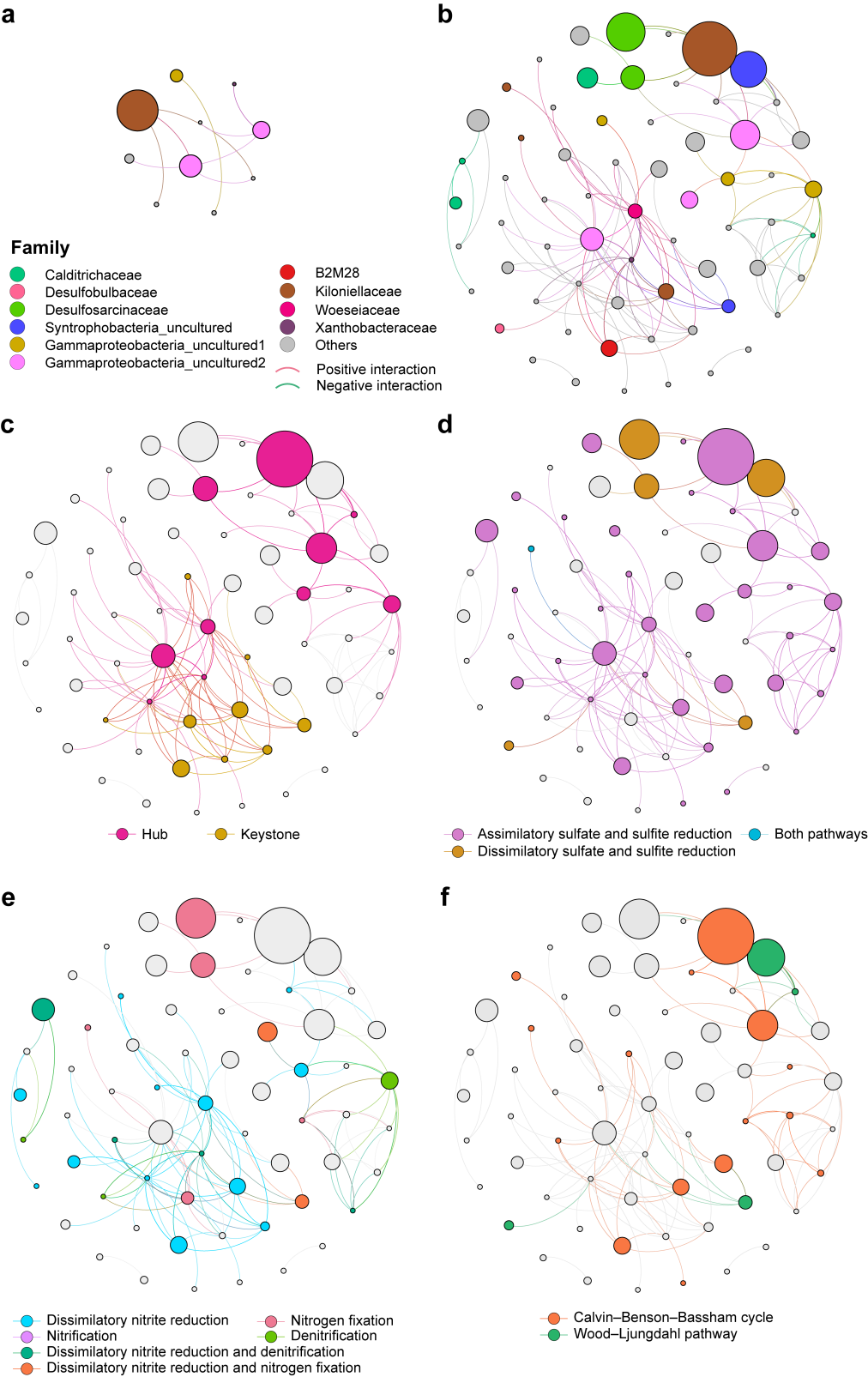


Fig. Sf3 Network of co-occurring OTUs in the Chinese mangrove sediment samples sequenced with three primers (338F-806R, 341F-806R and 515F-806R). Nodes of networks are colored according to phylum (a, b), role in the network (c), and the type of sulfur reduction process (d), nitrogen metabolisms (e) and carbon fixation (f) harbored by the OTUs. The connections were filtered with *r* > 0.6, *p* < 0.05 (a), and *r* > 0.5, *p* < 0.05 (b-f). Node size is proportional to the relative abundance of each OTU.
